# Predictors of Benefit from Immune Checkpoint Inhibitors in Urothelial Carcinoma of the Bladder: The Limits of PD-L1 and the Rise of Circulating Tumor DNA-Guided Adjuvant Therapy

**DOI:** 10.3390/cancers18213581

**Published:** 2026-11-07

**Authors:** Dorin Novacescu, Mihael Musta, Radu Caprariu, Cristina Stefania Dumitru, Alina Cristina Barb, Antonia Armega-Anghelescu, Radu Dragomir, Adelina Baloi, Silviu Latcu, Flaviu Lucian Petrica, Alin Cumpanas, Flavia Zara

**Affiliations:** 1 Department II of Microscopic Morphology, Victor Babes University of Medicine and Pharmacy Timisoara, E. Murgu Square, No. 2, 300041 Timisoara, Romania; novacescu.dorin@umft.ro (D.N.); cristina-stefania.dumitru@umft.ro (C.S.D.); toma.alina@umft.ro (A.C.B.);; 2 Bastion Policlinic, Mircea cel Batran Street, No. 122C, 300600 Timisoara, Romania; silviu.latcu@umft.ro (S.L.); flaviu.petrica@policlinicabastion.ro (F.L.P.); 3 Doctoral School, Victor Babes University of Medicine and Pharmacy Timisoara, E. Murgu Square, No. 2, 300041 Timisoara, Romania; 4 Department XV, Discipline of Urology, Victor Babes University of Medicine and Pharmacy Timisoara, E. Murgu Square, No. 2, 300041 Timisoara, Romania; cumpanas.alin@umft.ro; 5 Department XV, Discipline of Radiology and Medical Imaging, Victor Babes University of Medicine and Pharmacy Timisoara, E. Murgu Square, No. 2, 300041 Timisoara, Romania; 6 Department of Oncology, Victor Babes University of Medicine and Pharmacy Timisoara, E. Murgu Square, No. 2, 300041 Timisoara, Romania; radu.dragomir@umft.ro; 7 Department X—Surgery II, Discipline of Anesthesia and Intensive Care, Victor Babes University of Medicine and Pharmacy Timisoara, E. Murgu Square, No. 2, 300041 Timisoara, Romania; adelina.baloi@umft.ro

**Keywords:** urothelial carcinoma, bladder cancer, immune checkpoint inhibitors, PD-L1, circulating tumor DNA, molecular residual disease, predictive biomarkers, tumor mutational burden, atezolizumab, enfortumab vedotin

## Abstract

Immune checkpoint inhibitors (ICIs) are established across the treatment continuum of urothelial carcinoma of the bladder, yet most patients derive no durable benefit. This narrative review appraises candidate predictors of ICI benefit in bladder urothelial carcinoma, distinguishing prognostic from predictive biomarkers, applying the same three evidentiary standards to every biomarker, classifying the supporting studies by design and separating evidence from regulatory status. Programmed death-ligand 1 (PD-L1) immunohistochemistry meets the enrichment standard in three co-primary PD-L1-positive populations, in trials also positive overall, but not the predictive standard: its four registrational assays are not interchangeable; randomized comparisons show inconsistent subgroup effects, failed prespecified tests in IMvigor211 and DANUBE and no significant treatment-by-PD-L1 interaction; and the benefit of first-line enfortumab vedotin plus pembrolizumab is independent of combined positive score. Its remaining role is confined to label restrictions, chiefly European. Tumor mutational burden, molecular subtypes, expression signatures, DNA damage response and FGFR3 alterations remain investigational. Tumor-informed circulating tumor DNA (ctDNA) answers a different question: whether residual disease persists after cystectomy. After an exploratory signal in IMvigor010, the prospective enrichment trial IMvigor011 showed that ctDNA-positive patients derive disease-free and overall survival benefit from adjuvant atezolizumab; persistently ctDNA-negative patients were observed without randomization, so their 88% two-year disease-free survival does not exclude a benefit of treatment. This underpins US companion-diagnostic approval and a Category 1 guideline recommendation, not yet European authorization. Post-cystectomy ctDNA thus meets the same enrichment standard as PD-L1 and, like PD-L1, neither the predictive nor the strategy standard; what sets it apart is that the marker was necessary for the result, adjuvant atezolizumab having failed unselected, and that it directs whether to treat rather than which regimen; its role after perioperative immunotherapy is unresolved.

## Simple Summary

Immunotherapy has transformed the treatment of bladder cancer, but not every patient benefits, and treatment carries risks and costs. Doctors have therefore spent a decade searching for a test that identifies in advance who will benefit. The test used most widely measures a protein called PD-L1 in tumor tissue. In this review we examined the main randomized trials and found that PD-L1 testing has largely failed to do what was hoped. Different laboratory tests give different answers, and across trials PD-L1 did not consistently separate patients who benefit from those who do not. By contrast, a blood test that detects fragments of tumor DNA after the bladder is removed identifies patients whose cancer is likely to return. In a randomized trial, such patients lived longer when given immunotherapy. Patients whose test stayed negative were not treated and most remained free of cancer, although about one in nine relapsed within two years, and because they were not randomized the trial could not show whether treatment would have helped them. We summarize what clinicians should and should not test for today.

## 1. Introduction

Bladder cancer remains a major global health burden, with approximately 614,000 new cases and 220,000 deaths worldwide in 2022 according to GLOBOCAN estimates [1,2], approximately three-quarters of which occurred in males [2]. Urothelial carcinoma accounts for the overwhelming majority of cases in industrialized countries. For patients with muscle-invasive or metastatic disease, outcomes were for decades determined almost entirely by platinum-based chemotherapy, and the therapeutic landscape was static.

That situation changed abruptly with the introduction of immune checkpoint inhibitors (ICIs). Beginning with the single-arm IMvigor210 and CheckMate 275 studies and the randomized KEYNOTE-045 trial, agents targeting the programmed cell death protein 1 (PD-1)/programmed death-ligand 1 (PD-L1) axis demonstrated durable responses in a minority of patients with platinum-refractory disease [3,4,5], and the single-arm KEYNOTE-052 study extended this activity to the first-line treatment of cisplatin-ineligible patients [6]. ICIs are now embedded across the entire disease continuum: in Bacillus Calmette–Guérin (BCG)-unresponsive and, most recently, BCG-naive non-muscle-invasive bladder cancer (NMIBC), in the perioperative management of muscle-invasive bladder cancer (MIBC), both with chemotherapy and, since late 2025, with an antibody–drug conjugate (ADC), as adjuvant therapy after radical cystectomy, in combination with an ADC in the first line, and as switch-maintenance after platinum chemotherapy.

This success has been partial rather than universal. Objective response rates to ICI monotherapy in advanced urothelial carcinoma cluster in the 15–30% range [3,4,5,6], meaning that the majority of treated patients are exposed to immune-related toxicity, financial cost and the opportunity cost of forgone alternative therapy without deriving benefit. The clinical and economic case for a reliable predictive biomarker is therefore compelling, and the search for one has consumed a substantial share of translational effort in the field.

PD-L1 immunohistochemistry (IHC) dominated that search. Because PD-L1 is the direct ligand of the receptor being blocked, it possessed strong biological face validity, and it was adopted into trial design, stratification and regulatory labeling remarkably early—arguably before the evidence justified it. The subsequent history of PD-L1 in urothelial carcinoma is, we contend, a cautionary tale about the difference between a biologically plausible marker and a clinically actionable one.

Meanwhile, a biomarker with no direct mechanistic link to checkpoint biology has traveled further along the translational path than PD-L1 ever did. Tumor-informed circulating tumor DNA (ctDNA), used to detect molecular residual disease (MRD) after radical cystectomy, generated a prespecified exploratory signal in IMvigor010 [7], was tested prospectively in the randomized enrichment trial IMvigor011 [8], and has since supported a companion-diagnostic approval and a Category 1 guideline recommendation in the United States. It does so, however, by answering a different question: not which tumors are sensitive to checkpoint blockade, but which patients still harbor disease after surgery. Nor has it been shown to be predictive in the strict sense defined in Section 2.1; what has been shown is that treatment triggered by a positive test improves outcomes, the enrichment standard of Section 2.1, which PD-L1 has also met in three trials; the difference between the two biomarkers, and what it rests on, is the subject of Section 2 and Section 11.

The aim of this review is to appraise, rigorously and evenhandedly, the evidence supporting each class of candidate predictor of ICI benefit in urothelial carcinoma of the bladder, and to state plainly which biomarkers should influence clinical decision-making in 2026 and which should not. We classify each supporting study by design, apply the same evidentiary standard to every biomarker, and keep the level of evidence separate from regulatory and guideline status, which differs between jurisdictions (Section 2). We summarize the resulting biomarker landscape across the disease continuum. We restrict our scope to bladder-origin urothelial carcinoma, referring to upper tract disease only where trial populations were mixed and this materially affects interpretation.

## 2. Conceptual and Methodological Framework

### 2.1. Prognostic Versus Predictive Biomarkers

Much of the confusion in this literature stems from conflation of two distinct concepts. A prognostic biomarker informs the natural history of disease irrespective of the therapy administered; it identifies patients who will fare well or badly whatever is done. A predictive biomarker identifies patients who derive differential benefit from a specific intervention relative to a comparator [9,10].

The distinction is not semantic; it has a strict methodological consequence. Establishing that a biomarker is predictive requires a randomized controlled trial containing a non-ICI comparator arm, with the biomarker measured in both arms and a formal test of the treatment-by-biomarker interaction [11,12,13]; where a trial was not designed around the marker, a prospectively specified analysis of archived specimens from a randomized trial can provide strong evidence, provided that it is replicated [14]. A high response rate in a biomarker-positive subgroup of a single-arm trial demonstrates nothing about prediction; response rates to chemotherapy are also higher in prognostically favorable patients. Similarly, a subgroup hazard ratio that appears more impressive in biomarker-positive patients is not evidence of prediction unless the interaction test is significant and the analysis was prespecified and adequately powered. Throughout this review we therefore quote subgroup hazard ratios descriptively: a numerical difference between two subgroup estimates, in either direction, is not evidence of effect modification. Where a trial reports estimates for both biomarker strata but no interaction test, we have calculated an approximate test of interaction from the published hazard ratios and confidence intervals, as the ratio of the two hazard ratios with a standard error derived from the two intervals [15]. Such tests are post hoc, assume independence of the strata and are underpowered, because detecting an interaction of the same magnitude as a main effect requires roughly four times the sample size [16,17]; we report them to make explicit what the subgroup data can and cannot show, not as substitutes for prespecified analyses. For each trial the test is calculated for the endpoint that the trial reports by stratum, which is its primary endpoint (overall survival in the metastatic trials, disease-free survival in the adjuvant trials and event-free survival in NIAGARA); where an adjuvant trial also reports overall survival by stratum, both are given, so that the choice of endpoint does not favor either biomarker. Sixteen such tests are reported in this review (see Tables and Figures below). None was prespecified and no adjustment for multiplicity is made, so that under the null hypothesis about 0.8 of them would be expected to reach *p* < 0.05 by chance and a Bonferroni-corrected threshold would be about 0.003; the *p* values are therefore descriptive and are not used to rank the biomarkers against each other.

Trial design determines which of these questions can be answered [11,12,18]. A marker-stratified design randomizes both biomarker-positive and biomarker-negative patients and therefore permits an interaction test. An enrichment design randomizes only biomarker-positive patients: it can show that treatment works in that group, but it cannot show that treatment would fail in the group it excludes. A marker-strategy design randomizes patients between biomarker-guided and conventional management and tests the clinical utility of the strategy as a whole. The three designs answer three different questions, and the answers must not be interchanged. A marker-stratified design can show whether the treatment effect differs between marker-positive and marker-negative patients. An enrichment design can show that treatment is effective in the marker-positive population; it cannot show that selecting patients by the marker is better than treating all of them, and it cannot show whether marker-negative patients would also benefit, so it does not by itself justify withholding treatment from them. A marker-strategy design can show that marker-guided management improves outcomes, or spares treatment without worsening them, compared with unguided management, which is clinical utility in the strict sense. A trial that randomizes all patients but prespecifies an alpha-controlled comparison in the marker-positive population, the co-primary design used in several trials of PD-L1, contains an enrichment comparison embedded in a marker-stratified trial: the co-primary comparison tests efficacy in the marker-positive population, and the complementary stratum, where reported, is an exploratory subgroup unless an interaction test was prespecified. We use these terms throughout, because the two biomarkers at the center of this review were evaluated with different designs, and the difference determines what each can claim.

Three standards therefore run through this review, and each is applied in the same way to every biomarker. The first, the predictive standard, requires a significant treatment-by-biomarker interaction in a randomized comparison with a non-ICI control, prespecified and replicated. The second, the enrichment standard, requires a prospective randomized comparison with a non-ICI control, restricted to or prespecified for the marker-positive population, in which treatment improved outcomes; it establishes that treating marker-positive patients is effective and establishes nothing about marker-negative patients. The third, the strategy standard, requires a randomized comparison of marker-guided with unguided management showing that the guided strategy improves outcomes or spares treatment without worsening them; only this standard establishes that using the marker is better than not using it. The three must not be conflated. A biomarker can meet the second without meeting the first or the third; this, as Section 5 and Section 11 show, is the position of ctDNA, and it is also, for three agent-and-assay pairs, the position of PD-L1 (Section 3.4 and Section 3.5). What distinguishes the two is not the standard met but whether the marker was needed to reach the treatment decision (Section 11.2). All three standards are demanding, and to our knowledge no biomarker in urothelial carcinoma meets the first or the third. We apply them consistently throughout, to ctDNA as strictly as to PD-L1, and we state explicitly for each biomarker whether the supporting evidence derives from single-arm studies, from post hoc subgroup analyses or from prespecified randomized comparisons, and which standard, if any, it meets.

### 2.2. Classification of Evidence

To make these standards operational we classify every study cited in support of a biomarker claim by the design categories of Simon, Paik and Hayes [14], which were developed for this purpose. Category A is a prospective randomized trial designed to address the marker question, whether by a marker-stratified, enrichment or marker-strategy design; it yields level I evidence for the question that the design can answer and none for the questions it cannot (Section 2.1), so that a co-primary marker-positive comparison is category A for the enrichment question, whereas the complementary stratum of the same trial, reported as a subgroup without a prespecified biomarker analysis plan or a power calculation, is category D. Category B is a prospective trial not designed around the marker in which the marker analysis was nevertheless specified in writing before the assays were performed, used a validated assay and was performed on archived specimens from enough patients, representative of the trial, to give adequate statistical power; it yields level II evidence alone and level I when replicated in a second category B study. Category C is a prospective observational registry or cohort, and category D is a retrospective or post hoc analysis, including an unplanned subgroup analysis of a randomized trial; category C yields level II evidence when replicated and level III alone, category D yields level IV to V evidence, and both are hypothesis-generating. Single-arm trials, however large, cannot support a predictive claim, because the response rate in a marker-defined subgroup cannot be separated from the prognostic effect of the marker. We did not apply a formal risk-of-bias instrument, because the question for a predictive biomarker is one of design and pre-specification rather than of bias within studies; the design category of each cited analysis is stated explicitly, and where an analysis meets some but not all of the criteria of a category, we say which are unmet and grade it at the lower level (Section 5.2).

Clinical actionability is a separate matter. Whether a test should be used depends not only on the level of evidence but on regulatory authorization, the availability of a validated assay, guideline endorsement and reimbursement, all of which vary between jurisdictions. We report this implementation status separately from the level of evidence, so that a marker may be supported by level I evidence for one decision in one jurisdiction and be unavailable in another. Thus, later on in this review, we describe actionability as of October 2026, as opposed to a simple grading of evidence.

### 2.3. Evidence Sources

This is a narrative rather than a systematic review. In the absence of a reporting guideline for narrative reviews, we used the six items of the Scale for the Assessment of Narrative Review Articles (SANRA), a critical appraisal instrument rather than a reporting guideline, as a guide to quality during drafting, as its authors suggest [19]. We searched PubMed/MEDLINE and the proceedings of the American Society of Clinical Oncology (ASCO), the ASCO Genitourinary Cancers Symposium and the European Society for Medical Oncology from database inception to 30 October 2026 (date of the last search), combining terms for the disease, the intervention and each biomarker class (disease: “Urinary Bladder Neoplasms” [MeSH], “Carcinoma, Transitional Cell” [MeSH], “Non-Muscle Invasive Bladder Neoplasms” [MeSH], “urothelial carcinoma”, “bladder cancer”; intervention: “Immune Checkpoint Inhibitors” [MeSH], “immune checkpoint inhibitor”, “PD-1”, “PD-L1”, “CTLA-4”, atezolizumab, avelumab, durvalumab, nivolumab, pembrolizumab, ipilimumab, tremelimumab, sasanlimab, toripalimab, enfortumab vedotin, disitamab vedotin, sacituzumab govitecan; biomarkers: “B7-H1 Antigen” [MeSH], “PD-L1 expression”, SP142, SP263, 22C3, “combined positive score”; “tumor mutational burden”; “Circulating Tumor DNA” [MeSH], “ctDNA”, “molecular residual disease”; “Microsatellite Instability” [MeSH], “mismatch repair”, “DNA damage response”; “molecular subtype”, “gene expression signature”, “TGF-β”, “interferon-γ”; “Nectin-4”, “HER2”, “Trop-2”; “FGFR3”, erdafitinib; setting: adjuvant, neoadjuvant, perioperative, “non-muscle-invasive”, “BCG Vaccine” [MeSH]; terms within a block combined with “OR” and blocks combined with “AND”), and we screened the reference lists of retrieved articles and of current guidelines. These sources were supplemented by regulatory documents from the U.S. Food and Drug Administration (FDA) and the European Medicines Agency (EMA) and by current clinical practice guidelines; regulatory documents and guideline versions were last verified on 30 October 2026.

Eligibility and prioritization were defined before screening. For each biomarker we sought to include every randomized phase 3 trial of an ICI in bladder urothelial carcinoma with a non-ICI comparator that reported an outcome by biomarker status, without selection among them, together with the published biomarker analyses of those trials, so that the randomized evidence presented here is intended as a census rather than a selection (trials comparing one ICI-containing regimen with another cannot address prediction of ICI benefit and were not sought for that purpose); single-arm studies, retrospective cohorts and congress abstracts were included only where no randomized evidence existed, and congress abstracts are identified as such where they are cited and treated as provisional. The search was supplemented on 30 October 2026 by a check of the randomized trials presented at the European Society for Medical Oncology Congress 2026 (23–27 October 2026), which added three perioperative trials with PD-L1-stratified results (VOLGA, KEYNOTE-866 and CA017-078/ENERGIZE; Section 5.7 and Section 9), available at that date as congress presentations and, for VOLGA, a simultaneous publication. When studies conflicted, we did not choose between them on the basis of their direction; all were reported, each was placed in its design category (Section 2.2), and greater weight was given to the higher category, to prespecified over post hoc analyses, to full publications over abstracts and to replicated over single findings. Records were screened by two authors (M.M. and D.N.) and disagreements were resolved by discussion with a third (R.C.). Because selection was not governed by a registered protocol, selection bias cannot be excluded, and this is acknowledged as a limitation in Section 11.4. Where the literature is internally contradictory, we say so rather than selecting the more convenient dataset.

## 3. PD-L1 Immunohistochemistry

### 3.1. Four Assays, Four Scoring Algorithms

**Figure 1:**
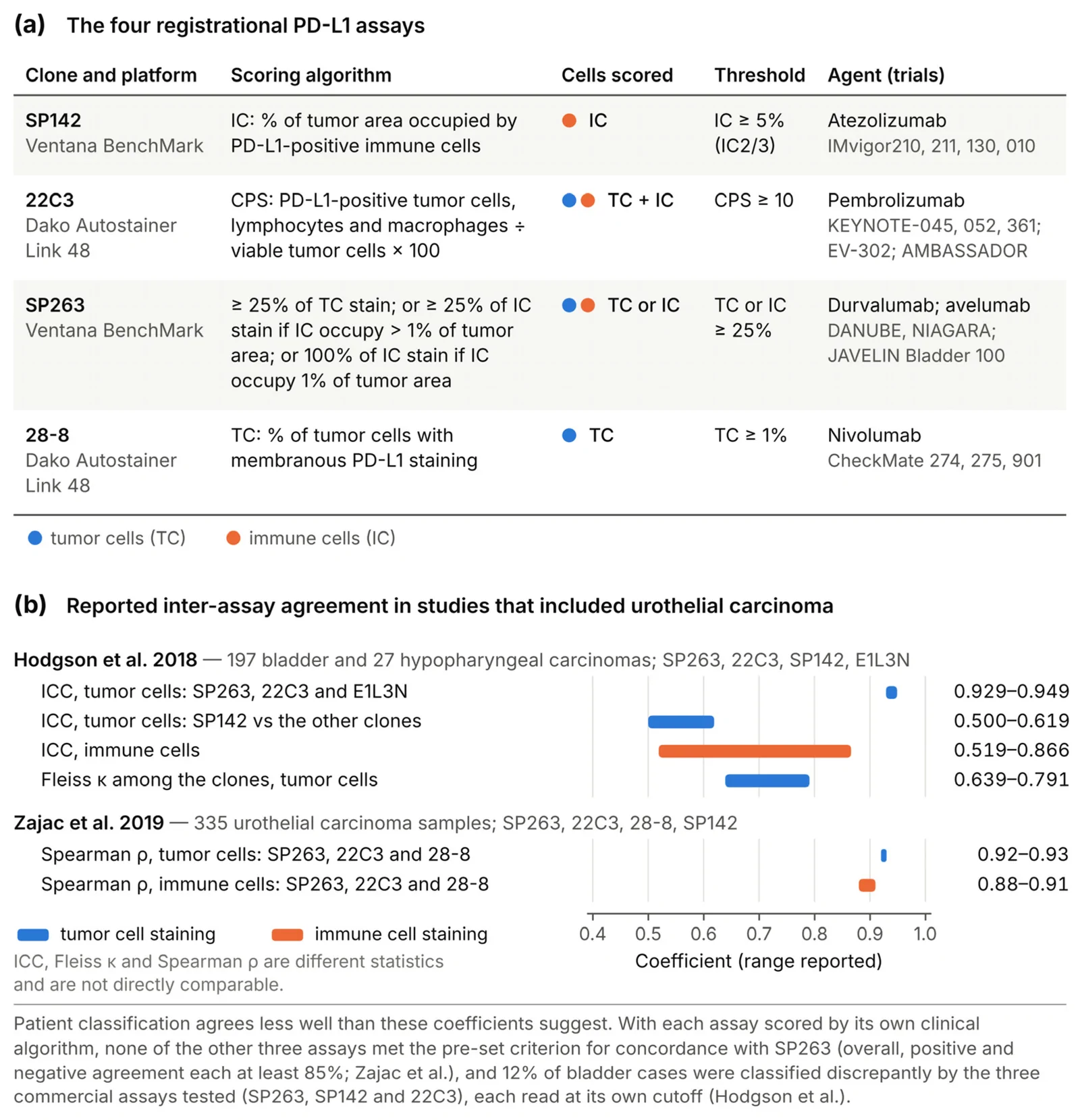
Heterogeneity of registrational PD-L1 immunohistochemistry assays in urothelial carcinoma. (**a**) The four registrational assays differ in antibody clone, scored cellular compartment and positivity threshold, and run on two staining platforms; assays scoring only immune cells (SP142) and those scoring only tumor cells (28-8) measure biologically distinct quantities. (**b**) Reported inter-assay agreement coefficients from concordance studies that included urothelial carcinoma. Each bar spans the range of coefficients reported; ICC, Fleiss κ and Spearman ρ are different statistics and are not directly comparable. Hodgson et al. did not test 28-8; their fourth clone, E1L3N, is not part of a registrational assay. Agreement among SP263, 22C3 and 28-8 for tumor cell staining is high, whereas SP142 agrees less well with the other clones and agreement for immune cell scoring is generally lower. In both panels, blue denotes tumor cells and orange denotes immune cells. Data from Hodgson et al. [20] and Zajac et al. [21]. CPS, combined positive score; IC, immune cells; ICC, intraclass correlation coefficient; PD-L1, programmed death-ligand 1; TC, tumor cells.

There is no single PD-L1 test. Four distinct registrational assays are in clinical use, each pairing a different antibody clone with one of two staining platforms and—critically—a different scoring algorithm applied to a different cellular compartment (see Table 1, Figure 1a).

The SP142 assay (Ventana) scores only tumor-infiltrating immune cells (IC), expressed as the percentage of tumor area occupied by PD-L1-positive immune cells, with IC2/3 denoting at least 5%. The 22C3 assay (Dako) uses the combined positive score (CPS), in which PD-L1-positive tumor cells, lymphocytes and macrophages are summed, divided by the number of viable tumor cells and multiplied by 100. The SP263 assay (Ventana) classifies a tumor as PD-L1-high if at least 25% of tumor cells stain or, where immune cells occupy more than 1% of the tumor area, if at least 25% of those immune cells stain; unlike SP142, its immune cell score is a percentage of the immune cells present, not of the tumor area. The 28-8 assay (Dako) scores membranous tumor cell staining, with 1% as the threshold relevant to adjuvant nivolumab.

These are not variations on a theme. An assay that counts only immune cells and one that counts only tumor cells are measuring biologically different quantities, and no amount of analytical harmonization can make them equivalent. The scoring algorithm, not merely the antibody, drives patient classification.

### 3.2. Analytical Concordance

Harmonization studies that included urothelial carcinoma consistently identify SP142 as an outlier (see Figure 1b). In 335 archival urothelial carcinoma samples assessed with all four assays, SP263, 22C3 and 28-8 correlated closely for both tumor cell staining (Spearman rank coefficients 0.92–0.93) and immune cell staining (0.88–0.91). Patient classification was another matter: the pre-set concordance criterion (overall, positive and negative percent agreement each at least 85%) was met only for 22C3 versus SP263, and only when the SP263 algorithm was applied to both (overall percent agreement 92.2%, positive percent agreement 86.4%, negative percent agreement 95.4%); when each assay was scored with its own clinical algorithm, none of the other three assays met the criterion for concordance with SP263 [21].

In a tissue-microarray study of bladder and hypopharyngeal carcinomas, intraclass correlation coefficients for tumor cell staining were excellent among SP263, 22C3 and E1L3N (0.929–0.949) but only moderate for SP142 (0.500–0.619); agreement for immune cell staining was generally lower (0.519–0.866), and 12% of the 197 bladder cases were classified discrepantly when the three commercial assays were each read with their own cutoff [20]. A dedicated tissue-microarray study of 124 invasive bladder urothelial carcinomas, analyzed per tissue core, reported overall percent agreement of 89.6% between 22C3 and SP263 using CPS, falling to 83.7% and 83.3% when SP263 and 22C3 respectively were compared with SP142 on immune cell scoring [22].

The practical implication is unambiguous: a patient classified as PD-L1-high by one registrational assay may be PD-L1-low by another. Any claim that PD-L1 predicts ICI benefit is therefore assay-specific and algorithm-specific, and cannot be generalized across agents. The same caution applies in reverse: the failure of one assay in one trial does not, by itself, condemn the others.

### 3.3. Preanalytical and Biological Heterogeneity

Analytical discordance is compounded by biological variability. PD-L1 expression is spatially heterogeneous within tumors and varies between specimen types: in matched transurethral resection, cystectomy and lymph node specimens from the same patients, agreement of PD-L1 status was only fair to moderate and exceeded the variability observed between assays, so that a small transurethral resection biopsy may not represent the cystectomy specimen [23]. Expression may also change over time and with intervening therapy: it was higher after neoadjuvant chemotherapy than before in matched specimens [24], and high immune cell expression agreed poorly between primary tumors and matched metastases [25], although paired pre- and post-treatment analyses in non-muscle-invasive disease did not show that intravesical BCG increases PD-L1 expression [26]. Interobserver reproducibility is imperfect for both cellular compartments—in a small multicenter round-robin study (14 cases read by three pathologists), agreement was moderate to substantial for immune cell scoring (κ 0.50–0.61) and only fair for tumor cell scoring (κ 0.26–0.35) [27]—and CPS—which requires the pathologist to enumerate several cell populations—is more demanding and less intuitive than a simple tumor proportion score.

### 3.4. Randomized Evidence

**Figure 2:**
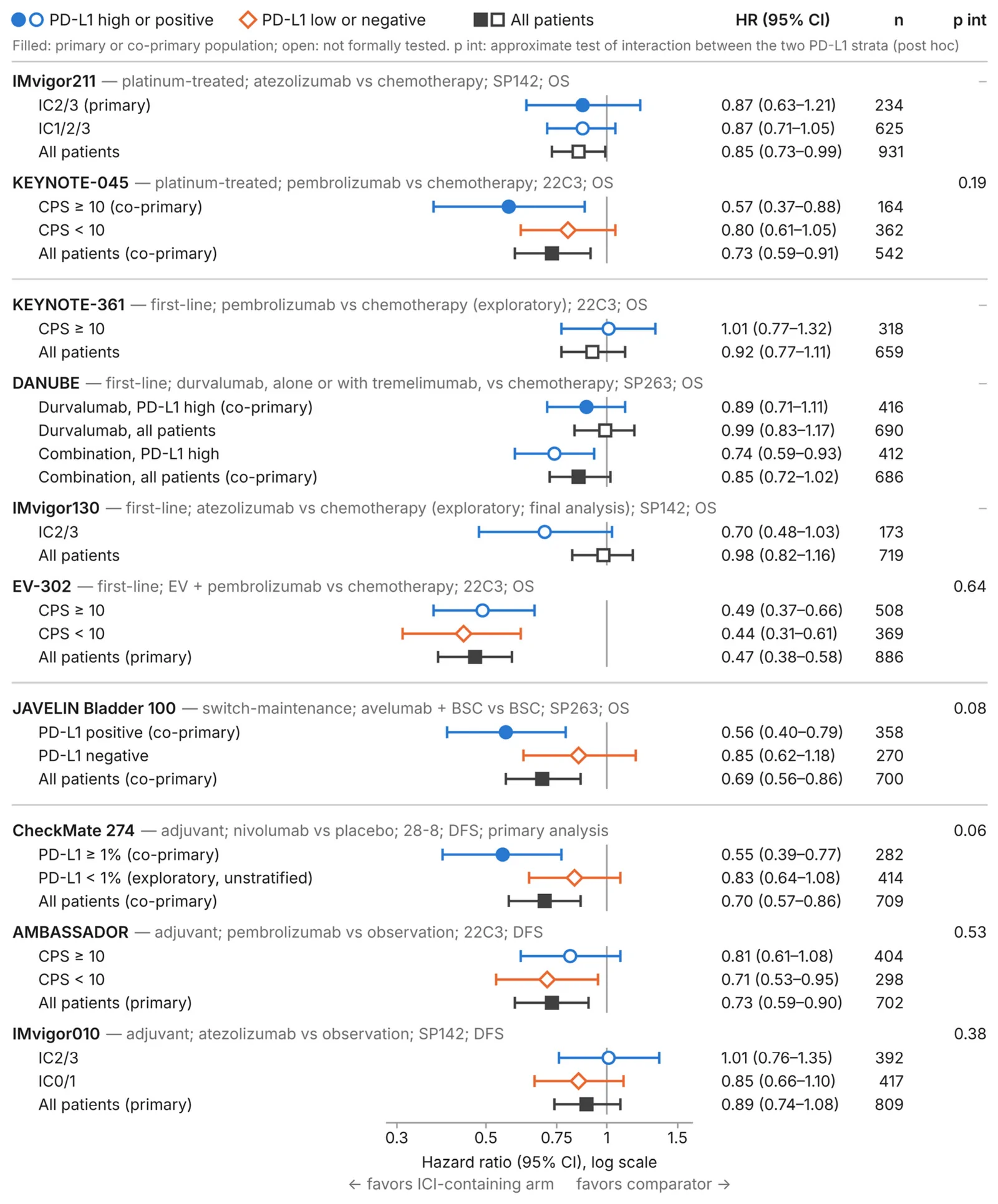
Treatment effect by PD-L1 status in the ten randomized phase 3 trials of immune checkpoint inhibitors in urothelial carcinoma whose primary publications report PD-L1-stratified hazard ratios; eight further randomized comparisons with PD-L1-stratified results, reported in supplementary material, in congress presentations or without stratum estimates, are summarized below. Hazard ratios (HR) and 95% confidence intervals (CI) are reproduced from the primary publication of each trial, except for IMvigor130 (final analysis); for CheckMate 274 all three estimates are taken from the primary analysis, so that the two PD-L1 strata are compared within the same analysis (95% confidence intervals as reported in the United States prescribing information; Section 3.5), and the 5-year estimates are given in the text. *n* is the number of patients in each comparison, and the symbols are explained in the key. For the six trials that report both strata, the right-hand column gives an approximate test of interaction calculated from the published estimates (the ratio of the two hazard ratios with its *p* value; Section 2.1); these tests are post hoc, underpowered and unadjusted for multiplicity, and none reaches conventional significance, in either direction. A numerically lower hazard ratio in the PD-L1-high or PD-L1-positive stratum (KEYNOTE-045, JAVELIN Bladder 100 and CheckMate 274) or in the complementary stratum (EV-302, AMBASSADOR and IMvigor010) is therefore not evidence of effect modification; in the other four trials the PD-L1-high population can be compared only with all patients, a comparison of overlapping populations that is not a test of effect modification. Trials used different assays and thresholds, so estimates are not directly comparable across studies. BSC, best supportive care; CPS, combined positive score; DFS, disease-free survival; EV, enfortumab vedotin; IC, immune cells (IC0/1, IC1/2/3 and IC2/3: PD-L1-positive immune cells in <5%, ≥1% and ≥5% of the tumor area); ICI, immune checkpoint inhibitor; OS, overall survival; PD-L1, programmed death-ligand 1; *p* int, approximate interaction *p* value. Data from [5,28,29,30,31,32,33,34,35,36,37,38].

The most direct randomized test of PD-L1 as a predictive biomarker (strictly, of the SP142 immune cell score) was IMvigor211 (see Figure 2), in which 931 patients with platinum-treated metastatic urothelial carcinoma were randomized to atezolizumab or investigator’s choice chemotherapy [28]. Overall survival was tested hierarchically, beginning in the PD-L1 IC2/3 population. In that prespecified primary population (*n* = 234), atezolizumab did not improve survival: median 11.1 versus 10.6 months, stratified hazard ratio (HR) 0.87 (95% confidence interval [CI] 0.63–1.21; *p* = 0.41). Because the hierarchy was broken at the first step, no formal statistical claim could be made for any other population.

The subgroup estimates must be read with the caution set out in Section 2.1. In the intention-to-treat population the HR was 0.85 (95% CI 0.73–0.99), and in the broader IC1/2/3 population (*n* = 625) it was 0.87 (95% CI 0.71–1.05), the same as the point estimate in the PD-L1-high group; but these are nested, overlapping populations, so their numerical similarity is not a test of effect modification, and because the complementary IC0/1 stratum was not reported, no interaction test, approximate or otherwise, is possible (see Figure 2 and Tables below). What the trial does show is that the enrichment hypothesis failed where it was tested, in the prespecified IC2/3 comparison. The medians show something different, a prognostic association: survival was longer in the IC2/3 population than in the intention-to-treat population in both arms (10.6 versus 8.0 months with chemotherapy; 11.1 versus 8.6 months with atezolizumab) [28]. Whether high expression by this assay also modified the relative effect of atezolizumab cannot be determined from the published data.

The failed hierarchy has two implications for the predictive claim. First, the enrichment hypothesis was refuted in the population chosen to maximize its chance of success: if PD-L1 by the SP142 assay identified patients with a larger relative benefit, the IC2/3 comparison should have been the easiest to win, and it was lost. Second, because the hierarchy stopped at the first step, the intention-to-treat estimate (HR 0.85, 95% CI 0.73–0.99) is formally descriptive, so the trial provides no statistically controlled evidence of benefit in any population; a biomarker-first design, adopted on the strength of single-arm response rates, cost the trial the opportunity to establish atezolizumab in the unselected population. The wide confidence interval in the IC2/3 population (0.63–1.21) means that a modest benefit there is not excluded, and the unusually long survival of the IC2/3 chemotherapy arm, together with subsequent therapies, may have diluted any difference [28]; but neither consideration rescues the predictive hypothesis, which required a larger effect in this population, not a smaller one.

Other randomized comparisons with chemotherapy give a mixed picture. In the second-line KEYNOTE-045 trial, which used the 22C3 assay and tested overall survival in co-primary populations, pembrolizumab improved survival over chemotherapy both in all patients (HR 0.73, 95% CI 0.59–0.91) and in those with CPS of at least 10 (HR 0.57, 95% CI 0.37–0.88); in the subgroup with CPS below 10 the HR was 0.80 (95% CI 0.61–1.05) [5]. By the standards of Section 2.1, the co-primary comparison meets the enrichment standard for pembrolizumab with the 22C3 assay; because the comparison in all patients was positive as well and the CPS < 10 subgroup was exploratory, it does not show that PD-L1-negative patients should be treated differently. Within the final analysis summarized by the European product information, the hazard ratios were 0.55 (95% CI 0.37–0.81) for CPS of at least 10 and 0.75 (95% CI 0.59–0.95) for CPS below 10, so that with longer follow-up the benefit in the PD-L1-low stratum also became significant [39]. In the first-line setting the pattern differs by trial. In KEYNOTE-361, exploratory analyses performed after the primary hypotheses had failed showed pembrolizumab monotherapy to be no better than chemotherapy either overall (HR 0.92, 95% CI 0.77–1.11) or in patients with CPS of at least 10 (HR 1.01, 95% CI 0.77–1.32) [32]. In DANUBE, durvalumab monotherapy did not improve survival over chemotherapy in the co-primary PD-L1-high population (SP263; HR 0.89, 95% CI 0.71–1.11) or in the intention-to-treat population (HR 0.99, 95% CI 0.83–1.17), whereas durvalumab plus tremelimumab had a lower hazard ratio in PD-L1-high patients (0.74, 95% CI 0.59–0.93) than in the intention-to-treat population (0.85, 95% CI 0.72–1.02), the second co-primary comparison, which was likewise not significant [33]. In IMvigor130, atezolizumab monotherapy was not superior to chemotherapy in the intention-to-treat population at the final analysis (HR 0.98, 95% CI 0.82–1.16), while the exploratory estimate in the IC2/3 subgroup was 0.70 (95% CI 0.48–1.03) [34,35]. None of these trials was designed around a formal treatment-by-PD-L1 interaction test, and a meta-analysis of six randomized trials found no difference in the relative effect of ICI versus non-ICI therapy between PD-L1-positive and PD-L1-negative subgroups (*p* = 0.93) [40]. Approximate tests of interaction derived from the published subgroup estimates (Section 2.1) are consistent with this: in KEYNOTE-045 the ratio of the hazard ratios in the CPS ≥ 10 and CPS < 10 subgroups was 0.71 (95% CI 0.43–1.19; *p* = 0.19), and the corresponding values for the other trials that report both strata are given below and in Figure 2. None reaches conventional significance; the three smallest *p* values (0.06 in CheckMate 274 and in CA017-078/ENERGIZE, 0.08 in JAVELIN Bladder 100) lie in the direction of greater benefit in PD-L1-positive patients, whereas in four trials (EV-302, AMBASSADOR, IMvigor010 and VOLGA) the point estimate favors the PD-L1-negative stratum, and all are post hoc, underpowered and unadjusted for multiplicity. The fairest reading is that PD-L1 carries an inconsistent, assay-dependent and at best modest predictive signal for ICI monotherapy, which is insufficient for selecting patients; the same data equally fail to show that PD-L1-negative patients derive no benefit.

These comparisons must also be read with the assays in mind, because “PD-L1-positive” is not one population. The registrational definitions classified about one-quarter of patients as positive in IMvigor211 (SP142, IC2/3; 234 of 931), about 30% in KEYNOTE-045 (22C3, CPS ≥ 10; 164 of 542), 40% in CheckMate 274 (28-8, tumor cells ≥ 1%; 282 of 709), about half in IMvigor010 (SP142, IC2/3; 392 of 809) and JAVELIN Bladder 100 (SP263; 358 of 700), and about 60% in DANUBE (SP263; 416 of 690), AMBASSADOR (22C3, CPS ≥ 10; 404 of 702) and EV-302 (22C3, CPS ≥ 10; 508 of 886) [5,28,29,30,33,36,37,38]. A threshold that selects a quarter of patients by immune-cell staining and one that selects three-fifths by a combined score are testing different hypotheses: an immune-cell score is more plausibly a marker of an inflamed and prognostically favorable microenvironment, whereas tumor-cell staining more directly reflects adaptive resistance along the axis being blocked, and the combined positive score mixes the two. Preanalytical differences add to this, because specimen type, archival age and intervening chemotherapy all alter PD-L1 status (Section 3.3). Three consequences follow. Cross-trial comparison of PD-L1 subgroup effects is confounded by definition, so that inconsistency between trials may reflect the assays as much as the biology; pooled estimates such as the meta-analysis cited above, whose authors state that they did not stratify the studies by assay because the platforms were considered to correlate well [40], assume an exchangeability between assays that the concordance data do not support; and the only valid comparison is the within-trial comparison of strata defined by the same assay, which is the level at which Figure 2, the interaction tests and the following summary tables are presented. The failure of a given assay in a given trial is therefore evidence about that assay and that agent, and the inconsistency across trials is itself a finding: no assay has produced a replicated interaction.

JAVELIN Bladder 100 is often cited as supporting PD-L1. Avelumab switch-maintenance improved overall survival both in all randomized patients (median 21.4 versus 14.3 months; HR 0.69, 95% CI 0.56–0.86; *p* = 0.001) and in the co-primary PD-L1-positive population (HR 0.56, 95% CI 0.40–0.79; *p* < 0.001) [29]. In the exploratory PD-L1-negative population the HR was 0.85 (95% CI 0.62–1.18). As in KEYNOTE-045, the co-primary comparison meets the enrichment standard of Section 2.1, here for avelumab with the SP263 assay, and as in KEYNOTE-045 the comparison in all randomized patients was positive as well. The directionally consistent benefit across strata, the absence of a significant treatment-by-PD-L1 interaction (the trial was designed around co-primary populations rather than an interaction test, and a later exploratory analysis found none: *p* = 0.08 at a median follow-up of 21 months and *p* = 0.35 at 38 months; our approximate test from the primary estimates gives the same value, *p* = 0.08) [41], and the clinical reality that withholding effective maintenance therapy from a PD-L1-negative patient would be indefensible, together mean that PD-L1 is not used to select candidates for avelumab maintenance in practice.

Regulatory history tells the same story of a signal real enough to act on but not robust enough to last. In mid-2018, early reviews by the data monitoring committees of the randomized KEYNOTE-361 and IMvigor130 trials found decreased survival with first-line pembrolizumab or atezolizumab monotherapy, compared with platinum-based chemotherapy, in patients whose tumors had low PD-L1 expression. The FDA and the EMA restricted both agents accordingly: in the United States to cisplatin-ineligible patients with PD-L1-high tumors or to patients ineligible for any platinum-containing chemotherapy regardless of PD-L1 status, and in the European Union to cisplatin-ineligible patients with PD-L1-high tumors [42,43]. This was randomized evidence that low PD-L1 expression identified patients who fared worse with monotherapy than with chemotherapy; but the final analyses of the same trials, summarized above, did not establish the converse, that high expression identifies patients who do better with monotherapy than with chemotherapy. In the United States, the post-platinum indications of durvalumab and atezolizumab and the first-line indication of atezolizumab were subsequently withdrawn [44], and in August 2021 the first-line pembrolizumab indication was converted to regular approval and confined to patients not eligible for any platinum-containing chemotherapy, irrespective of PD-L1 [45], so that, to our knowledge, no PD-L1 requirement still remains nowadays in any United States label for an ICI in urothelial carcinoma. By contrast, in the European Union, the PD-L1 restrictions on first-line pembrolizumab and atezolizumab monotherapy remain in the product information [39,46].

### 3.5. Where PD-L1 Retains Any Role

PD-L1 testing retains a role wherever a label requires it, and those labels are now mainly European. The clearest case is adjuvant nivolumab. CheckMate 274 prespecified PD-L1 at least 1% (28-8 assay) as a co-primary population alongside all randomized patients [36]. In the primary analysis, nivolumab improved disease-free survival in all randomized patients (median 20.8 versus 10.8 months; HR 0.70, 95% CI 0.57–0.86) and in patients with PD-L1 at least 1% (median not reached versus 8.4 months; HR 0.55, 95% CI 0.39–0.77), whereas in the exploratory subgroup with PD-L1 below 1% (*n* = 414, 58% of patients), the unstratified HR was 0.83 (95% CI 0.64–1.08) in the analysis reported in the United States prescribing information (0.82, 95% CI 0.63–1.06, in the trial’s own forest plot) [36,47]; an approximate test of interaction between the two strata gives *p* = 0.06 (see Tables below), and Figure 2 presents the three estimates from this single analysis, with the 95% confidence intervals given in the prescribing information, because the primary publication reports the two co-primary comparisons with 98.22% and 98.72% confidence intervals (0.55–0.90 and 0.35–0.85) [36,47]. At a five-year minimum follow-up the pattern persisted: median disease-free survival was 21.9 versus 11.0 months in all randomized patients (HR 0.74, 95% CI 0.61–0.90) and 55.5 versus 8.4 months in patients with PD-L1 at least 1% (HR 0.58, 95% CI 0.42–0.79), and overall survival, which did not cross the prespecified boundary for significance, favored nivolumab in both populations (HR 0.83, 95% CI 0.67–1.02, and HR 0.63, 95% CI 0.44–0.90, respectively); the estimate for PD-L1 below 1% at this analysis is given only in the supplementary appendix of that publication [31]. On the basis of the primary analysis, adjuvant nivolumab was authorized in the European Union only for tumors with tumor cell PD-L1 expression of at least 1% [48], whereas the FDA approved it without a PD-L1 restriction, reporting the exploratory PD-L1-negative estimate but drawing no restriction from it [49]. By the standards of Section 2.1, the co-primary comparison meets the enrichment standard for nivolumab with the 28-8 assay, the third such instance for PD-L1 after KEYNOTE-045 and JAVELIN Bladder 100 (see Tables below). The European restriction rests on the absence of established efficacy in the exploratory PD-L1-negative stratum, whose confidence interval includes a clinically relevant benefit, not on evidence that the stratum does not benefit.

The divergence between two regulators examining identical data has an identifiable basis in their methods rather than in the evidence. The FDA’s practice, when the primary analysis in all randomized patients is positive, is to approve the population that was randomized and to present subgroup results as exploratory; its approval announcement reports the PD-L1-negative hazard ratio of 0.83 and the immaturity of overall survival, and draws no restriction from either [47,49]. The Committee for Medicinal Products for Human Use (CHMP) works under a guideline on subgroups in confirmatory trials which states that an indication should not include subpopulations in which the evidence of efficacy is inadequate or a positive benefit–risk balance has not been concluded; that the credibility of a subgroup finding rests on prior specification, biological plausibility and replication rather than on an interaction test, which the guideline describes as lacking power and as an inadequate basis for decision-making on its own; and that a credible finding that efficacy is not established in a subgroup is to be reflected in the therapeutic indication [50]. PD-L1 status was a stratification factor and defined a co-primary population in CheckMate 274, so the subgroup met the guideline’s requirement of prior specification. The sponsor had applied for an unrestricted indication; after two rounds of questions the Committee adopted a positive opinion for the PD-L1-positive population only [48,51]. The same difference in approach produced the same divergence for adjuvant atezolizumab in non-small cell lung cancer, approved by the FDA for PD-L1 expression of at least 1% and by the EMA for at least 50% [46,52], and, in the opposite direction, the 2018 first-line restrictions described above, which the FDA relaxed for platinum-ineligible patients and the EMA did not [42,43]. The two agencies thus weigh the same subgroup evidence differently: the FDA asks whether the trial established benefit in the population that was randomized, the EMA whether benefit has been established in each population it licenses. Neither position rests on a demonstrated treatment-by-PD-L1 interaction, which CheckMate 274 did not test and which the approximate test above does not supply.

The second case is first-line monotherapy. The European product information still restricts pembrolizumab monotherapy in cisplatin-ineligible patients to tumors with CPS of at least 10, and atezolizumab monotherapy to tumors with PD-L1 expression of at least 5% [39,46]. The 2026 European Association of Urology (EAU) guideline accordingly lists adjuvant nivolumab and first-line monotherapy in cisplatin-ineligible patients as the current indications for PD-L1 testing, while noting that the predictive value of PD-L1 was not confirmed in the first-line phase 3 trials [53]. The population concerned is small and shrinking, because the same guideline recommends enfortumab vedotin plus pembrolizumab as the first-line standard and reserves single-agent ICI for patients who are not eligible for combination therapy; but for those patients in Europe, PD-L1 status still determines access to treatment. The practical corollary of EV-302 (Section 9) is that PD-L1 testing is no longer required to choose first-line therapy for a patient who can receive enfortumab vedotin plus pembrolizumab and should not delay it; testing is needed only for the minority who cannot, and only where the label requires it.

Even in the adjuvant setting the enrichment should not be over-read. CheckMate 274 reported that a higher probability of disease-free survival with nivolumab was observed regardless of PD-L1 status, the primary report includes no treatment-by-PD-L1 interaction test [36], and the other adjuvant trials do not reproduce the pattern. AMBASSADOR, testing adjuvant pembrolizumab against observation, found a disease-free survival benefit (median 29.6 versus 14.2 months; HR 0.73, 95% CI 0.59–0.90; *p* = 0.0027) that was, if anything, numerically larger in PD-L1-negative tumors (HR 0.71, 95% CI 0.53–0.95) than in PD-L1-positive tumors (CPS of at least 10; HR 0.81, 95% CI 0.61–1.08; approximate interaction *p* = 0.53), and the investigators concluded explicitly that PD-L1 was prognostic but not predictive and should not be used to select patients [37]. In IMvigor010, adjuvant atezolizumab was no more effective in IC2/3 tumors (unstratified HR 1.01, 95% CI 0.76–1.35) than in IC0/1 tumors (HR 0.85, 95% CI 0.66–1.10; approximate interaction *p* = 0.38), and immune cell PD-L1 expression was again a favorable prognostic factor [38]. Even the direction of the prognostic association appears inconsistent: in the placebo arm of CheckMate 274, median disease-free survival was numerically shorter in the PD-L1-positive population than in all randomized patients (8.4 versus 11.0 months) [31], whereas in the other two trials PD-L1 expression was associated with a better outcome. Three adjuvant trials using three assays thus give three different answers, none of which includes a significant interaction, and the trial in which PD-L1-high patients fared best (CheckMate 274) is also the one in which the complementary stratum came closest to no benefit; the pattern is compatible with a prognostic effect of the assay used, with a modest predictive effect confined to one agent and assay, or with chance.

## 4. Tumor Mutational Burden

Tumor mutational burden (TMB) has a coherent rationale: a higher somatic mutation count should generate more neoantigens and a more immunogenic tumor. Urothelial carcinoma is among the more highly mutated solid tumors, driven substantially by APOBEC mutational processes, making it a plausible candidate setting.

In the single-arm IMvigor210 study, higher TMB was associated with response to atezolizumab in both inflamed and immune-excluded tumors [54]. Exploratory analyses within randomized trials, which do include a non-ICI comparator, point in the same direction without settling the matter. In IMvigor211, the hazard ratio for death favored atezolizumab over chemotherapy in patients with TMB at or above the median (HR 0.68, 95% CI 0.51–0.90) but not in those below it (HR 1.00, 95% CI 0.75–1.32; approximate interaction *p* = 0.06, Section 2.1) [28]. In KEYNOTE-361, the overall survival hazard ratio for pembrolizumab versus chemotherapy was 0.69 (95% CI 0.45–1.04) in tumors with at least 175 mutations per exome and 0.92 (95% CI 0.73–1.16) below that threshold (approximate interaction *p* = 0.24) [55]; in KEYNOTE-045, pembrolizumab was favored over chemotherapy regardless of TMB, with a trend toward lower hazard ratios in the high-TMB subgroup [56]. Biomarker analyses of JAVELIN Bladder 100 and, in a congress abstract, of IMvigor130 likewise associated higher TMB with greater benefit from avelumab maintenance and from atezolizumab monotherapy, respectively [57,58]. In the ctDNA-positive subgroup of IMvigor010, patients whose tumors were also TMB-high (10 or more mutations per megabase; *n* = 69) showed a disease-free survival HR of 0.34 (95% CI 0.19–0.60), an exploratory finding in a small subgroup of an exploratory biomarker analysis [7]. None of these analyses was a prespecified, powered test of a treatment-by-TMB interaction, cutoffs and platforms differed between trials, and none has been prospectively replicated.

In June 2020 the FDA granted tissue-agnostic accelerated approval to pembrolizumab for TMB-high tumors (at least 10 mutations per megabase by FoundationOne CDx) on the basis of KEYNOTE-158, in which the objective response rate in the TMB-high cohort was 29% (95% CI 21–39) [59,60]. The essential caveat, insufficiently appreciated in practice, is that KEYNOTE-158 contained no urothelial carcinoma cohort. Its ten tumor types were anal, biliary, cervical, endometrial, salivary, thyroid and vulvar carcinoma, mesothelioma, neuroendocrine tumors and small-cell lung cancer.

The generalizability of the 10 mutations per megabase threshold across tumor types has been directly challenged. A pan-cancer analysis found that high TMB was associated with ICI response only in tumor types in which neoantigen load correlated with CD8 T-cell infiltration; bladder cancer, with melanoma and lung cancer, was among those types, and across them TMB-high tumors had a response rate of 39.8% [61]. That analysis therefore supports, rather than undermines, the biological relevance of TMB in urothelial carcinoma, although it rests on ICI-treated cohorts without comparators. Platform-dependent variation in TMB calculation, differing panel sizes and inconsistent cutoffs further limit portability.

We therefore regard TMB as the most plausible tissue-based candidate in bladder urothelial carcinoma, but not as an actionable one. No randomized trial in this disease has prospectively validated TMB as predictive, the exploratory signals from trials with a non-ICI comparator have not been confirmed by a formal interaction test, and the tissue-agnostic approval does not supply that evidence.

## 5. Circulating Tumor DNA and Molecular Residual Disease

### 5.1. Rationale and Assay Design

The approach validated in bladder cancer is a personalized, tumor-informed assay. Its feasibility was shown in a Danish cohort of 68 patients, in which patient-specific mutations tracked by ultra-deep sequencing of plasma detected metastatic relapse a median of 96 days before imaging [62]. In the commercial implementation used in the pivotal trials, whole-exome sequencing of the resected primary identifies patient-specific somatic variants, and a bespoke multiplex polymerase chain reaction next-generation sequencing panel tracks those variants in plasma. This design achieves far greater sensitivity at low variant allele frequencies than tumor-naive panels, which is essential in the MRD setting where the quantity of circulating tumor DNA may be vanishingly small. The post-cystectomy datasets from randomized trials discussed below (IMvigor010, IMvigor011, CheckMate 274 and NIAGARA) relied on the same commercial tumor-informed platform, with the exception of patients enrolled in mainland China in IMvigor011, who were tested with a molecular residual disease assay from a local laboratory (Tianjin Medical Laboratory, BGI) [8,63,64], and the evidence should not be assumed to transfer to other assay designs. The practical implications of the assay design, including its turnaround time and cost, are considered in Section 11.3.

Conceptually, ctDNA differs from every other biomarker considered in this review. It does not measure a mechanism of immune evasion; it measures the presence of residual cancer. Its behavior in the adjuvant setting may therefore be understood straightforwardly: patients without residual disease cannot benefit from systemic therapy directed at residual disease, whereas patients with it may. Two consequences follow. First, ctDNA positivity identifies who needs adjuvant therapy, not who is sensitive to checkpoint blockade: it would be expected to enrich for benefit from any effective adjuvant treatment, and it leaves open which ctDNA-positive patients will respond. Second, a negative test means that residual disease was not detected, not that it is absent, so the value of ctDNA as a negative selector depends on the sensitivity of the assay and on how often testing is repeated.

### 5.2. IMvigor010: The Prespecified Exploratory Signal

**Figure 3:**
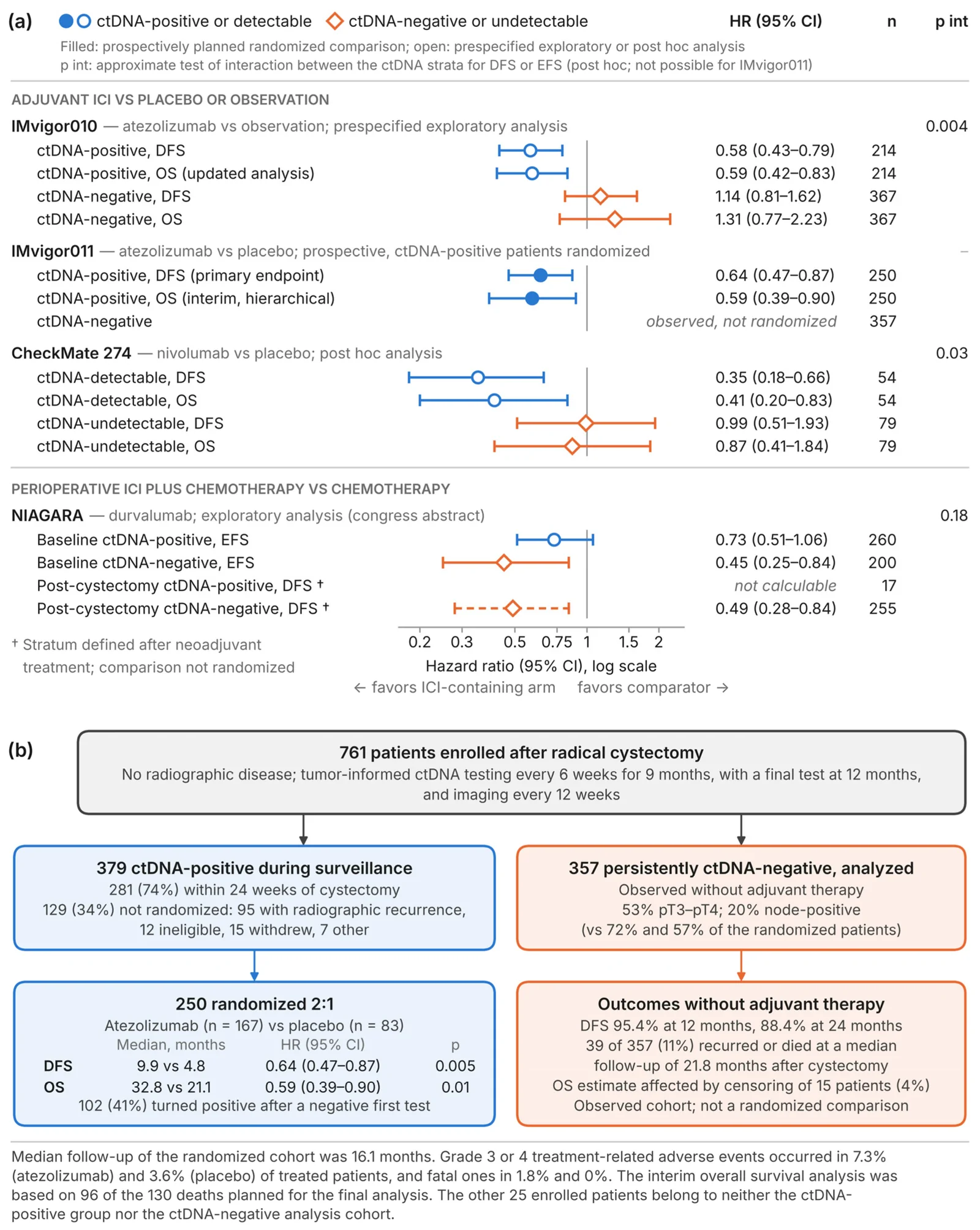
Circulating tumor DNA and benefit from adjuvant or perioperative immune checkpoint inhibitors. (**a**) Treatment effect by ctDNA status. Hazard ratios (HR) and 95% confidence intervals (CI) are reproduced from the cited sources; *n* is the number of patients in each analysis, and the symbols are explained in the key. The adjuvant estimates compare drug with placebo or observation within strata defined before treatment; disease-free and overall survival are shown for both strata where reported. The right-hand column gives an approximate test of interaction between the ctDNA strata for the disease-free or event-free survival estimates (Section 2.1): *p* = 0.004 for IMvigor010, *p* = 0.03 for CheckMate 274 and *p* = 0.18 for the baseline strata of NIAGARA; for overall survival, which IMvigor010 and CheckMate 274 also report by stratum, the corresponding values are *p* = 0.016 and *p* = 0.15 (Section 5.2 and Section 5.4). These tests are post hoc and unadjusted for multiplicity, and none is possible for IMvigor011. The NIAGARA estimates concern a perioperative regimen: its baseline strata were defined before treatment, whereas its post-cystectomy strata (^†^, dashed interval) were defined after neoadjuvant treatment that differed between arms, so the comparison within them is not randomized. (**b**) Design and principal outcomes of the IMvigor011 trial, to our knowledge the first randomized phase 3 trial of adjuvant therapy triggered by ctDNA positivity to show a survival benefit; overall survival was a key secondary endpoint tested hierarchically at an interim analysis (96 of 130 planned deaths), and the outcomes of the persistently ctDNA-negative cohort are those reported in the primary publication and the congress abstract. ctDNA, circulating tumor DNA; DFS, disease-free survival; EFS, event-free survival; ICI, immune checkpoint inhibitor; OS, overall survival; *p* int, approximate interaction *p* value. Data from [7,8,31,65,66,67,68].

IMvigor010 randomized patients with high-risk muscle-invasive urothelial carcinoma after resection to adjuvant atezolizumab or observation and was negative for its primary endpoint of disease-free survival (HR 0.89, 95% CI 0.74–1.08) [38]. A biomarker analysis whose statistical analysis plan was finalized before unblinding, but whose end points were exploratory, applied tumor-informed ctDNA testing to 581 evaluable patients (72% of the randomized population), of whom 214 (37%) were ctDNA-positive at cycle 1 day 1 [7] (see Figure 3a).

ctDNA positivity was powerfully prognostic: in the observation arm, the HR for disease-free survival comparing ctDNA-positive with ctDNA-negative patients was 6.3 (95% CI 4.45–8.92; *p* < 0.0001). More importantly, ctDNA-positive patients derived benefit from atezolizumab (disease-free survival HR 0.58, 95% CI 0.43–0.79, *p* = 0.0024; overall survival HR 0.59, 95% CI 0.41–0.86), whereas no difference between treatment arms was observed among ctDNA-negative patients (disease-free survival HR 1.14, 95% CI 0.81–1.62; overall survival HR 1.31, 95% CI 0.77–2.23). ctDNA clearance at week 6 occurred in 18% of the atezolizumab arm versus 4% of the observation arm [7].

The updated overall survival analysis confirmed the pattern: atezolizumab did not improve survival in the intention-to-treat population (HR 0.91, 95% CI 0.73–1.13), ctDNA positivity was strongly prognostic for overall survival in the observation arm (HR 6.3, 95% CI 4.3–9.3), and ctDNA positivity identified patients with an overall survival benefit favoring atezolizumab (HR 0.59, 95% CI 0.42–0.83) [65]. These analyses were nonetheless exploratory and hypothesis-generating. Measured against the criteria of category B in the classification of Section 2.2, the analysis meets three of them: the ctDNA objectives were specified in an analysis plan finalized before unblinding, the assay was validated, and the biomarker-evaluable population, 72% of those randomized with outcomes similar to the intention-to-treat population, exceeds the two-thirds that Simon and colleagues suggest. It does not meet the other two: no sample size was predetermined for the ctDNA study, so adequate power was not established in advance, and there is no replication in a second category B study (the CheckMate 274 analysis of Section 5.4 is post hoc). We therefore grade it as category B with criteria incompletely met, yielding at most level II evidence (see Tables below). Two points deserve emphasis. The confidence interval of the ctDNA-negative estimate (0.81–1.62) is compatible with a clinically relevant benefit as well as with harm, so the absence of an apparent effect in that stratum is not proof of no effect. On the other hand, applying to these data the same approximate interaction test that we apply to PD-L1 (Section 2.1), the ratio of the hazard ratios in the ctDNA-positive and ctDNA-negative strata was 0.51 (95% CI 0.32–0.81; *p* = 0.004) for disease-free survival and 0.45 (95% CI 0.24–0.86; *p* = 0.016) for overall survival in the primary ctDNA analysis [7] (*p* = 0.013 with the updated ctDNA-positive estimate [65]), a stronger signal of effect modification than any PD-L1 analysis has produced, although it is exploratory, unadjusted for multiplicity and requires confirmation in a trial that randomizes ctDNA-negative patients. A re-analysis of IMvigor010 samples with a second personalized assay, in which the tracked variants were selected from tissue comprehensive genomic profiling rather than whole-exome sequencing, reproduced the pattern in 396 patients (ctDNA-positive: disease-free survival HR 0.56, 95% CI 0.38–0.83; ctDNA-negative: HR 1.28, 95% CI 0.88–1.88) [69].

### 5.3. IMvigor011: A Prospective Randomized Enrichment Trial

IMvigor011 is, to our knowledge, the first randomized phase 3 trial to show a disease-free and overall survival benefit from adjuvant therapy triggered by ctDNA-detected molecular residual disease [8]. Other randomized trials of ctDNA-guided adjuvant management have been conducted in colorectal cancer: the marker-strategy trial DYNAMIC showed that ctDNA-guided management of stage II colon cancer reduced the use of adjuvant chemotherapy without compromising recurrence-free survival [70], whereas ALTAIR, which randomized patients who became ctDNA-positive after standard therapy to trifluridine/tipiracil or placebo, did not meet its primary disease-free survival endpoint (HR 0.79, 95% CI 0.60–1.05) [71]. In IMvigor011, patients with muscle-invasive bladder cancer and no radiographic evidence of disease entered surveillance 6 to 24 weeks after radical cystectomy and underwent tumor-informed ctDNA testing every 6 weeks for 9 months, with a final test at 12 months, and imaging every 12 weeks, for up to one year [8,64,67]. As seen in Figure 3b, of 761 patients enrolled, 250 eligible patients who tested ctDNA-positive were randomized 2:1 to atezolizumab 1680 mg or placebo every four weeks for up to 12 cycles; patients who remained persistently ctDNA-negative received no adjuvant therapy, and 357 of them formed the ctDNA-negative analysis cohort [8]. In design terms this is an enrichment trial: only ctDNA-positive patients were randomized, so the trial tests whether atezolizumab is effective in that group and cannot, by construction, test a treatment-by-ctDNA interaction (category A, level I evidence for the question it was designed to answer, and no evidence for the question it was not); in the terms of Section 2.1 it meets the enrichment standard yet, by construction, neither the predictive nor the strategy standard.

The flow of patients into the randomized cohort deserves attention. In all, 379 patients tested ctDNA-positive during surveillance, about three-quarters of them within 24 weeks of cystectomy, according to a congress abstract [67], but only 250 were randomized. Of the remaining 129, 95 were excluded because radiographic recurrence was already present (72 on investigator assessment and 23 on independent review), 12 did not meet other eligibility criteria, 15 withdrew and 7 were excluded for other reasons [8]. In those patients ctDNA positivity did not precede radiographic recurrence by long enough for adjuvant treatment to be given, and the efficacy results describe the roughly two-thirds of ctDNA-positive patients who were randomized.

At a median follow-up of 16.1 months, the primary endpoint, investigator-assessed disease-free survival among ctDNA-positive patients, was met: events had occurred in 112 of 167 patients (67%) assigned to atezolizumab and in 66 of 83 (80%) assigned to placebo, and median disease-free survival was 9.9 months (95% CI 7.2–12.7) versus 4.8 months (95% CI 4.1–8.3) (stratified HR 0.64, 95% CI 0.47–0.87; *p* = 0.005). Overall survival was the key secondary endpoint, tested hierarchically after disease-free survival to control the type I error, at an interim analysis performed at the time of the primary analysis: 60 patients (36%) in the atezolizumab arm and 36 (43%) in the placebo arm had died, median overall survival was 32.8 months (95% CI 27.7–not estimable) versus 21.1 months (95% CI 14.7–not estimable), and the HR was 0.59 (95% CI 0.39–0.90; *p* = 0.01), which crossed the prespecified Lan–DeMets (O’Brien–Fleming) boundary at that interim analysis (see Figure 3b) [8,64]. Because the overall survival analysis was an interim one, based on 96 of the 130 deaths planned for the final analysis (74%) in a trial with 60% power for this endpoint, the magnitude of the survival benefit may change with longer follow-up, although its statistical significance was established within the hierarchy. Twelve-month disease-free survival rates were 44.7% and 29.6%, and twelve-month overall survival rates 85.1% and 70.0%, respectively [68]. Disease-free survival was reported as generally consistent across subgroups, with the exception of PD-L1 status, a stratification factor, for which the benefit appeared larger in patients with PD-L1 immune-cell expression of at least 5%, in an analysis presented only in the supplementary appendix and without an interaction test [8]; because the trial compared an ICI with placebo, this PD-L1 subgroup is listed with the others (in Tables below). The benefit is real but partial: more than half of the ctDNA-positive patients assigned to atezolizumab had relapsed or died within a year.

Two features of the design deserve emphasis. First, about 41% of the randomized ctDNA-positive patients had converted from negative to positive during serial monitoring rather than testing positive at the first post-operative draw (102 of 250 patients; the other 148 were positive at the initial test). Single-timepoint testing would have missed a substantial proportion of the randomized patients, and the disease-free survival hazard ratios were similar whether positivity was present at the first test (unstratified HR 0.62, 95% CI 0.42–0.91) or emerged later (HR 0.66, 95% CI 0.40–1.10), in an analysis that was not prespecified, although the trial did not compare serial with single-timepoint testing [8]. Second, the persistently ctDNA-negative cohort—357 patients, of whom 53% had pT3–pT4 disease and 20% node-positive disease—had favorable outcomes without any adjuvant treatment: at a median follow-up of 21.8 months after cystectomy, 39 (11%) had recurred or died, and disease-free survival was 95.4% at the end of the one-year monitoring period and 88.4% at two years [8,68]; the corresponding overall survival estimate is affected by the censoring of 15 patients (4%) who recurred during monitoring and left the trial [8]. Three caveats apply to this cohort, and they govern every statement about it in this review. It was observed, not randomized, so the trial shows that these patients have a good prognosis without treatment, not that treatment would have added nothing; no estimate of a treatment effect in ctDNA-negative patients can be derived from IMvigor011 at all. It is defined by remaining negative throughout monitoring, a classification known only in retrospect: patients whose test turned positive left the cohort, and a patient had to remain alive, relapse-free and negative to stay in it, so its outcomes describe patients who never tested positive during monitoring, not everyone who is negative at a given draw, and they are flattered by this selection. And a negative result was not a guarantee: the estimated risk of relapse or death by two years was about 12% [8].

Toxicity is not trivial and must enter the discussion with patients. Grade 3 or 4 treatment-related adverse events occurred in 7.3% of atezolizumab recipients versus 3.6% of placebo recipients; treatment was discontinued for adverse events in 9.1% versus 3.6%; and fatal treatment-related adverse events occurred in three patients (1.8%) in the atezolizumab arm and none in the placebo arm [8].

### 5.4. Corroborating and Discordant Datasets

A post hoc exploratory ctDNA analysis of CheckMate 274 independently corroborates the IMvigor paradigm. Among 133 assessable patients, 54 (40.6%) had detectable baseline ctDNA after radical surgery. Median disease-free survival was 52.1 months in ctDNA-undetectable patients versus 5.0 months in ctDNA-detectable patients (HR 0.30, 95% CI 0.18–0.48), confirming the prognostic effect. Within the ctDNA-detectable subgroup, nivolumab improved disease-free survival (HR 0.35, 95% CI 0.18–0.66) and overall survival (HR 0.41, 95% CI 0.20–0.83) versus placebo, whereas among the 79 ctDNA-undetectable patients neither disease-free survival (HR 0.99, 95% CI 0.51–1.93) nor overall survival (HR 0.87, 95% CI 0.41–1.84) differed significantly between arms [31]; the approximate interaction test between the two strata gives a ratio of hazard ratios of 0.35 (95% CI 0.14–0.90; *p* = 0.03) for disease-free survival and 0.47 (95% CI 0.17–1.33; *p* = 0.15) for overall survival. Because both ctDNA strata were randomized, this analysis, like that of IMvigor010, approximates a marker-stratified comparison, and the two agree. But the analysis covers fewer than one-fifth of the randomized patients and was post hoc (category D), the overall survival test is not significant, the confidence intervals in the undetectable stratum are wide enough to include a substantial benefit, and the investigators state that further validation is required.

Single-arm prospective data point in the same direction. In the Danish single-arm phase 2 TOMBOLA trial, 192 patients treated with neoadjuvant chemotherapy and radical cystectomy underwent serial tumor-informed ctDNA testing, with atezolizumab initiated on ctDNA positivity irrespective of imaging; 104 of 178 assessable patients (58%) became ctDNA-positive within two years, 50 of the 84 patients (60%) who completed atezolizumab and were evaluable achieved the primary endpoint of molecular and radiographic complete response, and one-year recurrence-free survival was 97% in ctDNA-negative versus 76% in ctDNA-positive patients [72]. TOMBOLA used an academic tumor-informed assay rather than the commercial platform of the IMvigor program, which offers some reassurance regarding assay generalizability, but its single-arm design cannot establish a predictive effect.

A dataset from the perioperative setting complicates the picture and must be presented rather than minimized. In an exploratory analysis of NIAGARA, which tested perioperative durvalumab added to neoadjuvant gemcitabine–cisplatin, 462 of the 1063 randomized patients were biomarker-evaluable. ctDNA positivity fell from 57% at baseline to 22% before cystectomy and 9% (31 of 345) after cystectomy, and clearance between baseline and cystectomy was more frequent with durvalumab (41% versus 31%) [66]; in the oral presentation, clearance was strongly associated with event-free survival (HR 0.32, 95% CI 0.22–0.47, for patients who cleared ctDNA versus those who did not, which is a prognostic comparison and not a treatment effect). Two findings bear on the residual-disease paradigm. The event-free survival hazard ratio favored durvalumab in both baseline strata, numerically more so in baseline ctDNA-negative patients (HR 0.45, 95% CI 0.25–0.84) than in ctDNA-positive patients (HR 0.73, 95% CI 0.51–1.06). And among the patients analyzed for disease-free survival after cystectomy, those who were ctDNA-negative (*n* = 255) fared better in the durvalumab arm (HR 0.49, 95% CI 0.28–0.84), whereas those who were ctDNA-positive (9 and 8 patients in the two arms) were too few for a hazard ratio to be calculated [66]. A subsequent analysis combining urinary tumor DNA with plasma ctDNA before cystectomy found that 24-month event-free survival was highest in dual-negative patients (*n* = 72; 90%, 95% CI 83–97), intermediate in urinary-positive/plasma-negative patients (*n* = 62; 75%, 95% CI 65–87) and lowest in dual-positive patients (*n* = 33; 55%, 95% CI 41–75), in a biomarker-evaluable population of 265 patients [73]. Both analyses are available only as congress abstracts and presentations.

How should this be reconciled with the adjuvant data? The first point is methodological. Post-cystectomy ctDNA status in NIAGARA was measured after randomization and after neoadjuvant treatment that differed between the arms, so that it is a post-randomization variable influenced by the treatment under study. Comparing arms within strata defined in this way is not a randomized comparison: durvalumab increased clearance (41% versus 31%), so the ctDNA-negative stratum of the durvalumab arm contains patients who would have remained positive without durvalumab and whose underlying prognosis differs from that of the ctDNA-negative patients in the control arm, and the two strata are therefore not exchangeable. The direction of the resulting bias is not even predictable from first principles, because the stratum in the durvalumab arm is at once enriched for patients converted by effective treatment and for patients with a worse baseline prognosis; what can be said is that the hazard ratio of 0.49 in post-cystectomy ctDNA-negative patients conflates the carried-forward effect of the neoadjuvant durvalumab already received, the effect of the adjuvant durvalumab that followed, and this selection. It therefore cannot show that continuing durvalumab after surgery helps patients in whom no residual disease is detectable, and a landmark analysis restricted to patients negative at cystectomy would not repair it, because the conditioning variable would still be post-treatment; only strata defined before randomization, or a principal-stratum analysis, would. The second point is statistical: the post-cystectomy ctDNA-positive group was far too small for any estimate. The third is clinical: patients in the durvalumab arm of NIAGARA had already received an ICI with neoadjuvant chemotherapy before the post-cystectomy measurement, whereas IMvigor010 and IMvigor011 tested adjuvant ICI in a largely ICI-naive population. The baseline strata, which were defined before treatment, are interpretable: the point estimates favored the perioperative regimen in both, with no evidence of interaction (approximate *p* = 0.18), so a negative ctDNA test at baseline does not identify patients who derive no benefit from perioperative immunochemotherapy, and a negative test after surgery cannot yet be taken to do so. The honest summary is that the ctDNA-guided paradigm is supported for adjuvant ICI in ICI-naive patients after cystectomy, and is not validated as a rule for de-escalating perioperative immunochemotherapy; NIAGARA shows that the question of whether ctDNA negativity identifies patients who can forgo an ICI has a different answer in different settings, which is itself a reason not to generalize the IMvigor011 result beyond the setting in which it was obtained.

### 5.5. ctDNA Dynamics in Metastatic Disease

In the metastatic setting the evidence is considerably less mature. An exploratory analysis of KEYNOTE-361 evaluated pre- and post-treatment ctDNA in 130 pembrolizumab-treated and 130 chemotherapy-treated patients [74]. Baseline ctDNA levels were associated with clinical outcomes for pembrolizumab but not for chemotherapy. Chemotherapy induced larger early ctDNA decreases than pembrolizumab, yet changes with pembrolizumab were more strongly associated with long-term outcomes and were enriched among patients with higher TMB and PD-L1. Critically, tumor-informed ctDNA change metrics did not show statistically significant independent value for explaining overall survival beyond radiographic change by Response Evaluation Criteria in Solid Tumors (RECIST) version 1.1 when jointly modeled. Short-term ctDNA reduction is therefore not a treatment-independent surrogate, and ctDNA dynamics should not currently be used to switch or discontinue therapy outside a clinical trial.

### 5.6. Regulatory and Guideline Status

On 15 May 2026 the FDA approved atezolizumab and atezolizumab/hyaluronidase-tqjs as adjuvant treatments for adults with muscle-invasive bladder cancer after cystectomy who have ctDNA molecular residual disease as determined by an FDA-authorized test, and concurrently approved Signatera CDx as the companion diagnostic [75]. The approval announcement specifies that patients with negative test results should continue serial testing until a positive result or completion of the 12-month testing window—a direct translation of the IMvigor011 serial-monitoring design.

The National Comprehensive Cancer Network (NCCN) subsequently updated its Bladder Cancer guidelines (in version 2.2026, retained in version 3.2026 of 6 August 2026) to recommend consideration of ctDNA-MRD testing for risk stratification and to determine the use of adjuvant immunotherapy after cystectomy in patients who have not received previous checkpoint inhibitor treatment, using an FDA-approved personalized, tumor-informed multiplex polymerase chain reaction next-generation sequencing assay, with a category 1 recommendation for atezolizumab in patients who test ctDNA-MRD-positive within one year of cystectomy, listed in the “other recommended” tier of adjuvant options both for patients who did not receive neoadjuvant chemotherapy and for those who did, in whom nivolumab is the preferred option [76]. The same guideline, which can list options beyond the current labels, still carries first-line atezolizumab monotherapy for cisplatin-ineligible patients whose tumors express PD-L1 (SP142, immune cells ≥ 5%) as a category 2B option “useful in certain circumstances”, although the corresponding United States indication has been withdrawn [44,76].

The position in Europe is different. At the time of writing (24 October 2026), ctDNA-guided adjuvant atezolizumab is not authorized in the European Union: the product information for atezolizumab contains no adjuvant or ctDNA-based urothelial indication [46]. The 2026 EAU guideline grades the statement that ctDNA-guided adjuvant atezolizumab improves disease-free and overall survival as level 1b evidence and describes ctDNA as a promising prognostic and predictive biomarker (level 2b), but it makes no recommendation on ctDNA testing; its adjuvant recommendations, both rated strong, remain cisplatin-based chemotherapy and nivolumab, the latter with the note that the EMA approval is confined to tumor cell PD-L1 expression of at least 1% [53]. For European clinicians the practical consequences are that the strategy tested in IMvigor011 is not yet available within label, that adjuvant nivolumab, restricted to PD-L1-positive tumors, remains the only authorized adjuvant immunotherapy for pathologically high-risk patients, and that access to tumor-informed testing, its turnaround time (the first result requires sequencing of the resected tumor) and its reimbursement can be expected to vary between health systems. A cost-effectiveness analysis based on IMvigor011, from the perspective of a US payer, found the ctDNA-guided strategy to be dominant over a five-year horizon [77]; whether this holds in European health systems is unknown, and the logistics, cost and consequences of testing are discussed in Section 11.3. The ctDNA evidence discussed in Section 5.2, Section 5.3, Section 5.4 and Section 5.5 is summarized in Table 2.

### 5.7. ctDNA in the Era of Perioperative Immunotherapy

The population in which ctDNA-guided adjuvant atezolizumab was tested is contracting, because perioperative regimens containing an ICI have now improved survival in four published randomized phase 3 trials. In NIAGARA, durvalumab added to neoadjuvant gemcitabine–cisplatin and continued after cystectomy improved event-free survival (HR 0.68, 95% CI 0.56–0.82) and overall survival (HR 0.75, 95% CI 0.59–0.93) [78]. In KEYNOTE-905/EV-303, perioperative enfortumab vedotin plus pembrolizumab improved event-free survival (HR 0.40, 95% CI 0.28–0.57) and overall survival (HR 0.50, 95% CI 0.33–0.74) compared with cystectomy alone in patients who were ineligible for, or declined, cisplatin [79]; in KEYNOTE-B15/EV-304, the same regimen improved event-free survival (HR 0.53, 95% CI 0.41–0.70) and overall survival (HR 0.65, 95% CI 0.48–0.89) compared with neoadjuvant gemcitabine–cisplatin in cisplatin-eligible patients [80]. The FDA approved perioperative durvalumab in March 2025 [81] and perioperative enfortumab vedotin plus pembrolizumab for cisplatin-ineligible patients in November 2025, extending the latter to all cystectomy candidates in July 2026 [82,83]. In the European Union, perioperative durvalumab is authorized [84], and perioperative enfortumab vedotin plus pembrolizumab, authorized for cisplatin-ineligible patients in June 2026 [39], was extended to all patients with resectable disease regardless of cisplatin eligibility on 21 October 2026 [85]. The fourth trial, VOLGA, first announced by press release [86] and granted priority review by the FDA on 25 September 2026, with a decision anticipated in the fourth quarter of 2026 [87], was presented and published on 24 October 2026: in patients who were ineligible for, or declined, cisplatin, perioperative durvalumab with neoadjuvant enfortumab vedotin improved event-free survival (HR 0.51, 95% CI 0.38–0.67) and overall survival (HR 0.58, 95% CI 0.40–0.83) compared with cystectomy alone, whereas the addition of tremelimumab to that regimen did not significantly improve overall survival at the interim analysis (HR 0.77, 95% CI 0.54–1.07) [88]. Two trials presented at the same congress, both available only as congress abstracts at the time of writing, show that the benefit of adding a PD-1 or PD-L1 inhibitor to neoadjuvant gemcitabine–cisplatin is not uniform across trials. In cisplatin-eligible patients, perioperative pembrolizumab with gemcitabine–cisplatin in KEYNOTE-866 (event-free survival HR 0.88, 95% CI 0.71–1.10; overall survival HR 1.00, 95% CI 0.79–1.27) [89] and perioperative nivolumab with gemcitabine–cisplatin in CA017-078/ENERGIZE (event-free survival HR 0.94, 95.74% CI 0.71–1.25; pathological complete response 39.1% versus 29.6%, not significant) [90] both failed their primary endpoints, in contrast to durvalumab in NIAGARA.

These regimens change the question that ctDNA is asked. A patient who reaches cystectomy after perioperative immunotherapy is no longer ICI-naive and falls outside the NCCN recommendation; IMvigor011, whose eligibility criteria were framed around neoadjuvant chemotherapy or no neoadjuvant treatment [8], provides no direct evidence for such a patient, who will usually already be scheduled to continue the ICI after surgery. For such patients the relevant uses of ctDNA are different and untested: whether clearance of ctDNA before or after cystectomy can identify patients in whom the adjuvant component may be shortened or omitted; and whether persistent positivity should prompt a change in treatment rather than continuation of the same agent. The NIAGARA analyses discussed in Section 5.4 indicate that ctDNA remains prognostic in this setting but answer neither question, and to our knowledge no ctDNA analysis of the enfortumab vedotin plus pembrolizumab trials has yet been reported. As perioperative regimens are adopted, the proportion of post-cystectomy patients to whom the IMvigor011 result applies directly will fall; it will remain relevant mainly for patients who proceed to cystectomy without neoadjuvant immunotherapy, whether by choice, ineligibility or lack of access.

### 5.8. Unresolved Questions

Several important questions remain open. The optimal frequency and duration of serial testing, including beyond 12 months, are undefined; the six-weekly schedule used in IMvigor011 is resource-intensive, and about one-third of the patients who tested ctDNA-positive under it were not randomized. ctDNA-guided atezolizumab has not been compared with the existing standard of adjuvant nivolumab given on pathological risk alone, so it is unknown whether treating at molecular relapse is as good as, better than or worse than treating all high-risk patients at once. For a ctDNA-negative patient with pT3–pT4 or node-positive disease who qualifies for nivolumab, observation rests on the favorable outcome of the IMvigor011 negative cohort and on the lack of an apparent ICI effect in ctDNA-negative patients in the prespecified exploratory analysis of IMvigor010 (category B, criteria incompletely met) and the post hoc analysis of CheckMate 274 (category D), whose confidence intervals do not exclude a moderate benefit, not on a prospectively planned randomized comparison; the interaction signals in those analyses (Section 5.2 and Section 5.4) await confirmation. Establishing whether such patients can be safely observed in routine practice, as opposed to within a trial, will require longer follow-up of the ctDNA-negative cohort than is yet available. The predictors of benefit within the ctDNA-positive group, in which most patients still relapse, are unknown, although exploratory analyses of IMvigor010 implicate TMB and immune gene-expression signatures (Section 4 and Section 6.2). The ongoing Alliance A032103 (MODERN) trial (NCT05987241), which randomizes ctDNA-positive patients to nivolumab with or without relatlimab and ctDNA-negative patients to nivolumab or surveillance with treatment on conversion to positivity, will address both escalation and de-escalation questions [91]. Finally, the randomized evidence comes almost entirely from one commercial tumor-informed platform; although TOMBOLA used a different tumor-informed assay [72] and an alternative personalized assay reproduced the IMvigor010 findings [69], formal equivalence of other assays has not been demonstrated [63], and the ESMO recommendations on ctDNA assays require analytical and clinical validation of each assay for each intended use before adoption [92]. The IMvigor011 result is therefore evidence for the assay, schedule and setting used in the trial, not for ctDNA testing in general; an assay with lower sensitivity would enlarge the “negative” group and dilute its favorable prognosis, and one with lower specificity would treat patients without residual disease.

## 6. Molecular Subtypes and Tumor Microenvironment Signatures

### 6.1. Consensus Molecular Classification

Transcriptomic classification of muscle-invasive bladder cancer converged in 2020 on a consensus taxonomy derived from 1750 tumors, reconciling the earlier Cancer Genome Atlas (TCGA) and Lund schemes [93,94]. Six classes were defined: luminal papillary, luminal non-specified, luminal unstable, stroma-rich, basal/squamous and neuroendocrine-like.

Associations with ICI outcome have been reported but are not consistent across studies. IMvigor210 described response enrichment in TCGA luminal cluster II [3], a subtype later termed luminal-infiltrated [94], whereas CheckMate 275 reported the highest response rate in the basal 1 cluster [4], and a re-analysis of IMvigor210 under the consensus classification found responders enriched among luminal non-specified, luminal unstable and neuroendocrine-like tumors [93]. No subtyping scheme has been validated prospectively as predictive, no standardized clinical-grade assay exists, and classifier assignment is unstable at the margins between adjacent classes. Molecular subtyping is a valuable research tool and should not currently inform treatment selection.

### 6.2. Immune Phenotypes and Gene Expression Signatures

The most mechanistically informative analysis in this disease derived from IMvigor210, in which tumors were classified as immune-inflamed, immune-excluded or immune-desert [54]. Response to atezolizumab was associated with a CD8 T-effector phenotype and with high TMB, whereas non-response was associated with a transforming growth factor beta (TGF-β) signature in fibroblasts. The associations were phenotype-dependent: the CD8 T-effector score was associated with response only in inflamed tumors (*p* = 0.042), the fibroblast TGF-β signature was associated with non-response only in excluded tumors (*p* = 0.0066), while TMB was associated with response in both excluded and inflamed phenotypes (*p* = 0.00009 and *p* = 0.00258, respectively). In murine models, combined TGF-β and PD-L1 blockade provoked T-cell infiltration into tumor parenchyma and tumor regression.

A notable convergence with the ctDNA literature deserves mention: in ctDNA-positive patients treated with atezolizumab in IMvigor010, non-relapse was associated with immune response signatures and basal-squamous features, whereas relapse was associated with angiogenesis and fibroblast TGF-β signatures [7]. This is biologically satisfying but remains exploratory. No gene-expression signature is a standardized, validated, clinically deployable predictive assay in bladder cancer.

Neoadjuvant trials, in which the whole tumor is available before and after treatment, add a further layer of inconsistency. In PURE-01, a pathological complete response to neoadjuvant pembrolizumab was more frequent in tumors with CPS of at least 10 than in those with lower scores (54.3% versus 13.3%) and was associated with TMB [95]. In ABACUS, by contrast, response to neoadjuvant atezolizumab correlated with pre-existing activated T cells but not with TMB, and TGF-β and fibroblast activation were linked to resistance [96]. With combined blockade of cytotoxic T-lymphocyte-associated protein 4 (CTLA-4) and PD-1 or PD-L1, complete response in NABUCCO was independent of baseline CD8 T-cell presence and T-effector signatures [97], and in a second trial response was associated with the density of tertiary lymphoid structures rather than with PD-L1 expression, TMB or DNA damage response alterations [98]. All four were single-arm studies of fewer than 100 patients. They are valuable for generating hypotheses, the tertiary lymphoid structure among them, but they illustrate how readily tissue biomarkers change rank from one cohort and regimen to the next.

## 7. Genomic Alterations

### 7.1. DNA Damage Response Genes

Alterations in DNA damage response and repair genes, most frequently in ATM, POLE, BRCA2, ERCC2, FANCA and MSH6, were associated with higher response rates and longer survival in 60 patients with metastatic urothelial carcinoma treated with atezolizumab or nivolumab, with a gradient in response rate from wild-type tumors (19%) through alterations of unknown significance (54%) to known or likely deleterious alterations (80%) [99]. A second retrospective series of 53 patients found a higher response rate in tumors with three or more such alterations than in those with fewer (80% versus 24%), but no significant association between the number of alterations and overall survival [100]. These findings are retrospective, derived from ICI-treated cohorts without a non-ICI comparator, and are confounded by the well-established association between DNA damage response alterations and platinum sensitivity. They cannot support a predictive claim.

### 7.2. FGFR3 and the Immune-Cold Hypothesis

A widely repeated hypothesis holds that FGFR3-altered, luminal-papillary tumors are T-cell depleted and consequently less responsive to ICIs; it derives from transcriptomic analyses in which FGFR3 mutation and pathway activation were enriched among non-T-cell-inflamed tumors [93,101]. This proposition has clinical consequences, because it could be used to justify withholding immunotherapy from patients with FGFR3 alterations.

The question has two parts that the available evidence does not separate. FGFR3 alterations are prognostic in a general sense: they are concentrated in luminal-papillary, lower-stage and lower-grade disease and are associated with better outcomes in muscle-invasive cohorts irrespective of treatment [93,94]. Whether they are also predictive of reduced benefit from ICI relative to chemotherapy can be answered only by a randomized comparison with FGFR3 status measured in both arms, and none has been reported. The evidence that exists is of three kinds, none of which can make the distinction. Analyses of ICI-treated trial cohorts (IMvigor210 and CheckMate 275) found no significant difference in response rates between FGFR3-mutant and wild-type tumors [102], and a real-world cohort analysis reached a similar conclusion [103]; but a cohort without a comparator arm cannot tell whether an equal response rate reflects equal sensitivity or a better prognosis offsetting lower sensitivity, and reported associations across other retrospective series are inconsistent and lack interaction testing. Randomized data bear on the question only indirectly: in cohort 2 of the phase 3 THOR trial, which enrolled previously treated patients with FGFR-altered tumors who had not received an anti-PD-1 or anti-PD-L1 agent, erdafitinib did not improve overall survival over pembrolizumab (median 10.9 versus 11.1 months; HR 1.18, 95% CI 0.92–1.51), and responses to pembrolizumab, although less frequent (21.6% versus 40.0%), were more durable [104]; because every patient in the trial had an FGFR alteration, it shows that an ICI is active in this population but says nothing about benefit relative to FGFR3-wild-type patients. The conclusion is that neither a prognostic nor a predictive effect of FGFR3 status on ICI benefit has been established, that the two cannot be distinguished with the data available, and that the immune-cold hypothesis, however attractive mechanistically, has outrun its evidence base. FGFR3 testing is important because it identifies candidates for erdafitinib; it is not a reason to deny checkpoint inhibition.

### 7.3. Mismatch Repair Deficiency and Microsatellite Instability

Mismatch repair deficiency and high microsatellite instability (dMMR/MSI-H) define the second genomic context, alongside high TMB (Section 4), in which an ICI has tissue-agnostic approval from the FDA: pembrolizumab received accelerated approval for dMMR/MSI-H solid tumors in 2017 on the basis of pooled single-arm trials [105], which was converted to regular approval in March 2023 [106], whereas its approval for TMB-high tumors was still an accelerated approval in October 2026 [107]. The phenotype is uncommon in urothelial carcinoma and is concentrated in upper tract tumors. In an institutional sequencing series reported as a congress abstract, 26 of 1194 patients (2%) had MSI-H tumors, 77% of which arose in the upper tract, and nine of the ten patients treated with an ICI had complete or near-complete responses [108]. For the rare patient with bladder urothelial carcinoma and a dMMR/MSI-H tumor, this is an approved indication for an ICI in the United States and a prompt for germline assessment for Lynch syndrome; but the evidence is single-arm, for urothelial carcinoma specifically, and the phenotype identifies too few patients to bear on the wider problem of selection.

## 8. Clinical and Peripheral Blood Indices

Established clinical variables—Eastern Cooperative Oncology Group performance status, the presence of liver metastases, hemoglobin concentration, and the composite Bellmunt risk score derived from them [109]—together with laboratory indices such as the neutrophil-to-lymphocyte ratio and lactate dehydrogenase [110], are robustly associated with outcome in urothelial carcinoma. They are, however, overwhelmingly prognostic. They identify patients who do poorly on any therapy, including chemotherapy. The two references cited are prognostic models derived from treated cohorts, not tests of effect modification, and the randomized trials of ICI versus chemotherapy or best supportive care report these variables only as forest-plot subgroups with overlapping confidence intervals and without interaction tests [5,28,29]; there is therefore no convincing randomized evidence that any of them identifies differential benefit from ICIs relative to a comparator, although this is an absence of evidence rather than evidence of absence. In EV-302, for example, the relative benefit of enfortumab vedotin plus pembrolizumab over chemotherapy at the primary analysis was the same in patients with and without liver metastases (overall survival HR 0.47, 95% CI 0.32–0.71, and 0.47, 95% CI 0.36–0.61, respectively) [30]; because the experimental arm combined an ICI with an antibody–drug conjugate, this shows that liver metastasis does not modify the effect of the combination, not that it is uninformative for an ICI given alone. These indices are appropriate for prognostic counseling and trial stratification but should not be used to select for or against immunotherapy.

## 9. Biomarkers in the Antibody–Drug Conjugate Era

The therapeutic landscape shifted decisively with EV-302/KEYNOTE-A39, in which 886 patients with previously untreated locally advanced or metastatic urothelial carcinoma were randomized to enfortumab vedotin plus pembrolizumab or platinum-based chemotherapy, without biomarker preselection [30]. Median progression-free survival was 12.5 versus 6.3 months (HR 0.45, 95% CI 0.38–0.54) and median overall survival 31.5 versus 16.1 months (HR 0.47, 95% CI 0.38–0.58), with confirmed objective response rates of 67.7% versus 44.4%. At 2.5 years of median follow-up, these results were maintained (progression-free survival HR 0.48, 95% CI 0.41–0.57; overall survival HR 0.51, 95% CI 0.43–0.61) [111].

For the present discussion, the biomarker finding is as consequential as the efficacy result. PD-L1 was a stratification factor, and benefit was independent of it: the overall survival hazard ratio was 0.49 (95% CI 0.37–0.66) in patients with CPS at least 10 and 0.44 (95% CI 0.31–0.61) in those with CPS below 10 (approximate interaction *p* = 0.64) [30]; at 2.5 years the corresponding estimates were 0.55 (95% CI 0.43–0.70) and 0.47 (95% CI 0.36–0.62) [111]. The two estimates are similar. Because the regimen combines an ICI with an ADC and the comparator is chemotherapy, this result cannot show whether PD-L1 predicts the contribution of the ICI; what it does establish is the clinically decisive point that PD-L1 does not identify patients who should be denied, or preferentially given, the combination. When the first-line standard of care produces a near-halving of mortality irrespective of PD-L1, the biomarker has no role in selecting patients for that regimen. Three practical consequences follow for PD-L1 testing. It is not required before starting first-line enfortumab vedotin plus pembrolizumab and should not delay it. It remains necessary only for patients who are ineligible for the combination, for example because of pre-existing neuropathy, poorly controlled diabetes or poor performance status, and who are being considered for ICI monotherapy in a jurisdiction whose label requires it, which in 2026 means the European Union (Section 3.5). And because the result does not change management elsewhere, reflex PD-L1 testing of all urothelial carcinomas is no longer justified, which also bears on cost (Section 11.3). The EV-302 result does not show that PD-L1 is uninformative about the contribution of pembrolizumab within the combination, because there was no enfortumab vedotin-alone arm; it shows that the information, if any, is not clinically usable.

The same pattern recurs in the other contemporary phase 3 trials. In CheckMate 901, nivolumab added to gemcitabine–cisplatin improved overall survival (HR 0.78, 95% CI 0.63–0.96) and progression-free survival (HR 0.72, 95% CI 0.59–0.88) in cisplatin-eligible patients, with benefit irrespective of PD-L1 according to the investigators (overall survival HR 0.75, 95% CI 0.53–1.06, in the 36% of patients with tumor cell PD-L1 expression of at least 1%) [112]. In NIAGARA, perioperative durvalumab added to neoadjuvant chemotherapy improved event-free and overall survival (Section 5.7), with benefit consistent across subgroups, including those defined by PD-L1 expression (high in 777 of the 1063 patients, low or none in 286); notably, the investigators reported inconsistencies among the central laboratories in the prevalence of PD-L1 expression on immune cells with the TC/IC 25% algorithm, and therefore re-analyzed event-free survival by tumor-cell thresholds of 1% and 25%, with consistent results [78]. The perioperative enfortumab vedotin plus pembrolizumab trials likewise reported event-free and overall survival benefit consistent across PD-L1 CPS subgroups, with the stratum estimates confined to their forest plots and no interaction test [79,80], as did VOLGA for durvalumab with enfortumab vedotin (event-free survival HR 0.58, 95% CI 0.39–0.86, in PD-L1-high and 0.46, 95% CI 0.31–0.67, in PD-L1-low or negative tumors; approximate interaction *p* = 0.41) [88]. In the two trials that added a PD-1 inhibitor to neoadjuvant gemcitabine–cisplatin and failed their primary endpoints, both reported as congress abstracts, PD-L1 did not identify a subgroup with significant benefit either: in KEYNOTE-866 the event-free survival hazard ratio was 0.87 (95% CI 0.65–1.19) for CPS ≥ 10 and 0.94 (95% CI 0.68–1.28) for CPS < 10 (approximate interaction *p* = 0.73) [89], and in CA017-078/ENERGIZE it was 0.69 (95% CI 0.45–1.04) for PD-L1 ≥ 1% and 1.17 (95% CI 0.81–1.70) for PD-L1 < 1% (*p* = 0.06), a difference in the direction of greater benefit in PD-L1-positive tumors that, like the CheckMate 274 and JAVELIN Bladder 100 values, is post hoc, unadjusted and unconfirmed [90]. In the other randomization of CheckMate 901, nivolumab plus ipilimumab did not significantly improve overall survival over gemcitabine–carboplatin in cisplatin-ineligible patients (HR 0.79, 98.27% CI 0.61–1.01; *p* = 0.0245 against a boundary of 0.0173), nor in the co-primary population with tumor PD-L1 expression of at least 1% (HR 0.87; *p* = 0.36), a second failure, after DANUBE, of a PD-L1-selected co-primary comparison [113]. Across an entire generation of randomized trials, positive and negative, PD-L1 has repeatedly failed to identify a subgroup that should be treated differently (see Table 3).

**Figure 4:**
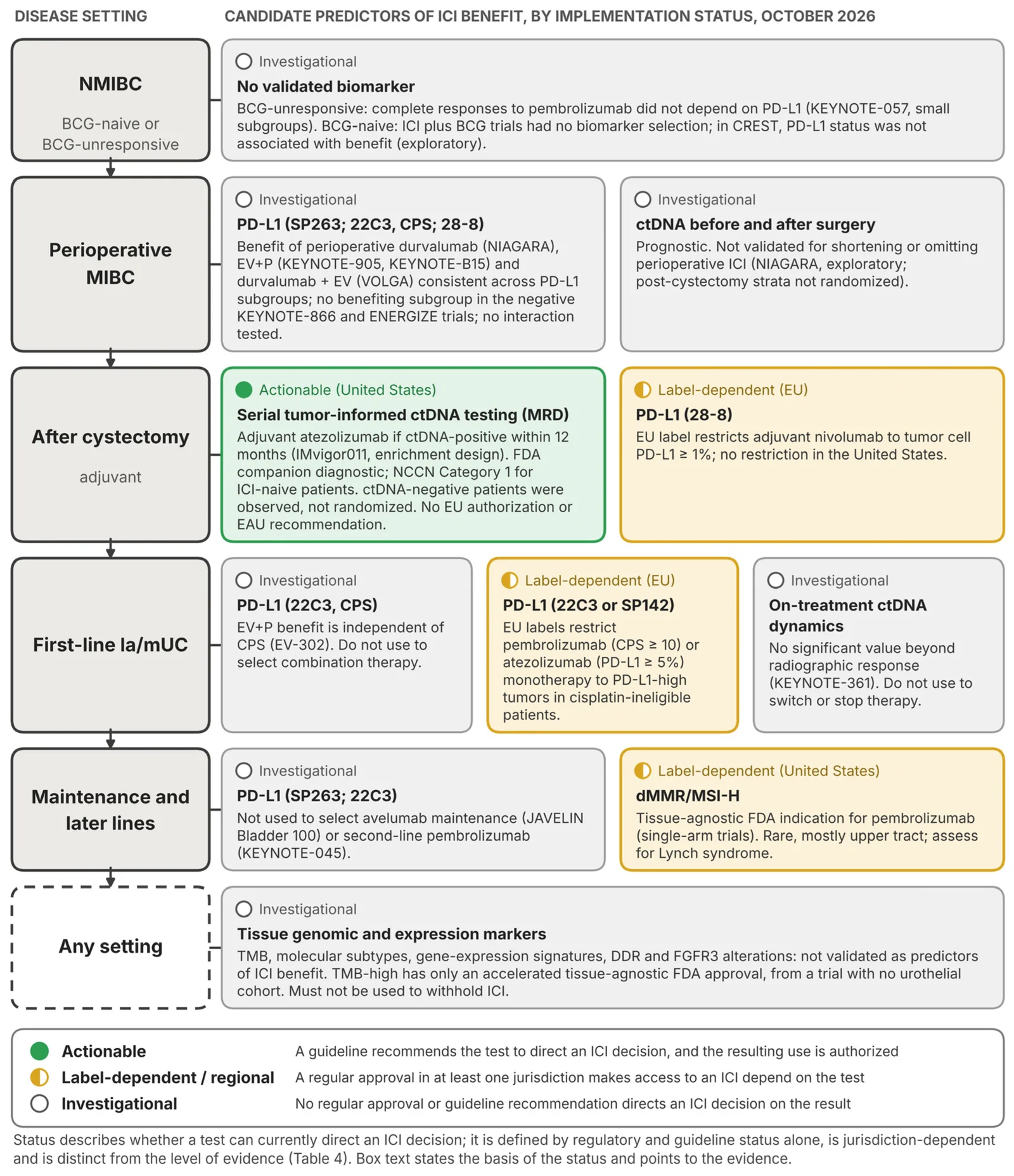
Candidate predictors of benefit from immune checkpoint inhibitors (ICI) across the disease continuum of bladder urothelial carcinoma, color-coded by implementation status (clinical actionability) as of October 2026. The tiers describe whether a test can currently direct an ICI decision, which depends on regulatory authorization, companion-diagnostic availability and guideline endorsement and is therefore jurisdiction-dependent; they are not a grading of the evidence, which is given by design category in Table 2, Table 3 and Table 4 and in the text (Section 2.2). The three tiers are defined in the key by implementation criteria alone, actionable (a guideline recommends the test to direct an ICI decision and the resulting use is authorized), label-dependent/regional (a regular approval in at least one jurisdiction makes access to an ICI depend on the test) and investigational (neither applies), and correspond to the implementation-status column of Table 4; the text in each box states the basis of the status and points to the evidence discussed in the text. Disease settings run from early (top) to late; the last row holds markers studied in several settings. Tiers and their symbols are defined in the key; PD-L1 is tiered separately in each setting. Only post-cystectomy tumor-informed ctDNA has both a companion-diagnostic approval and a guideline recommendation directing an ICI decision, so far in the United States only; even there, the randomized evidence concerns ctDNA-positive patients, and the observation of ctDNA-negative patients rests on an observed cohort. PD-L1 retains a limited role confined to label restrictions, chiefly European: adjuvant nivolumab, and first-line pembrolizumab or atezolizumab monotherapy in cisplatin-ineligible patients. BCG, Bacillus Calmette–Guérin; CPS, combined positive score; ctDNA, circulating tumor DNA; DDR, DNA damage response; dMMR, mismatch repair deficiency; EAU, European Association of Urology; EU, European Union; EV+P, enfortumab vedotin plus pembrolizumab; FDA, U.S. Food and Drug Administration; la/mUC, locally advanced or metastatic urothelial carcinoma; MIBC, muscle-invasive bladder cancer; MRD, molecular residual disease; MSI-H, high microsatellite instability; NCCN, National Comprehensive Cancer Network; NMIBC, non-muscle-invasive bladder cancer; PD-L1, programmed death-ligand 1; TMB, tumor mutational burden.

Combining ICIs with ADCs also shifts attention to the biomarkers of the ADC target, and here too, target expression has proved a weak guide. Nectin-4 protein expression did not predict benefit from enfortumab vedotin plus pembrolizumab in an exploratory analysis of EV-302, reported as a congress abstract [114]. Amplification of NECTIN4, present in about one-quarter of metastatic urothelial carcinomas, was associated with a 96% response rate to single-agent enfortumab vedotin, against 32% without amplification, in a retrospective multicenter series [115], and in a real-world cohort high NECTIN4 RNA expression was associated with longer survival on enfortumab vedotin plus pembrolizumab but was not significantly associated with outcome on pembrolizumab alone [116]; neither finding has been tested prospectively. For human epidermal growth factor receptor 2 (HER2), the randomized evidence comes from a biomarker-selected population: in tumors with HER2 immunohistochemistry scores of 1+ to 3+, disitamab vedotin plus toripalimab improved progression-free survival (HR 0.36, 95% CI 0.28–0.46) and overall survival (HR 0.54, 95% CI 0.41–0.73) over chemotherapy [117], and the 2026 EAU guideline now carries a weak recommendation for HER2 immunohistochemistry [53]. For TROP2, expression was nearly universal and did not predict the activity of sacituzumab govitecan [118]. None of these markers has been shown to predict benefit from the ICI component; they are relevant here because they will increasingly determine which combination a patient receives. The resulting biomarker landscape across the disease continuum is summarized in Figure 4.

**Table 3:** Randomized phase 3 trials of immune checkpoint inhibitors in bladder urothelial carcinoma with reported PD-L1-stratified outcomes.

Trial	Setting and Comparison	Assay	Overall Result	PD-L1 Subgroup Behavior
IMvigor211 [28]	Platinum-treated metastatic; atezolizumab vs. chemotherapy	SP142	OS HR 0.85 (0.73–0.99) in ITT	Failed prespecified IC2/3 primary endpoint (HR 0.87, 0.63–1.21); IC1/2/3 HR 0.87 (0.71–1.05; in appendix); IC0/1 not reported, so no interaction test.
KEYNOTE-045 [5]	Platinum-treated metastatic; pembrolizumab vs. chemotherapy	22C3	OS HR 0.73 (0.59–0.91)	CPS ≥ 10 (co-primary) HR 0.57 (0.37–0.88); CPS < 10 HR 0.80 (0.61–1.05); *p* int = 0.19.
KEYNOTE-361 [32]	First-line; pembrolizumab vs. chemotherapy (exploratory comparison)	22C3	OS HR 0.92 (0.77–1.11)	CPS ≥ 10 HR 1.01 (0.77–1.32); no enrichment.
DANUBE [33]	First-line; durvalumab ± tremelimumab vs. chemotherapy	SP263	OS HR in ITT: 0.85 (0.72–1.02) for the combination (co-primary); 0.99 (0.83–1.17) for durvalumab	Durvalumab in PD-L1-high (co-primary) HR 0.89 (0.71–1.11); combination in PD-L1-high HR 0.74 (0.59–0.93).
IMvigor130 [34,35]	First-line; atezolizumab vs. chemotherapy (exploratory comparison)	SP142	OS HR 0.98 (0.82–1.16) at final analysis	IC2/3 HR 0.70 (0.48–1.03).
JAVELIN Bladder 100 [29]	Switch-maintenance; avelumab + BSC vs. BSC	SP263	OS HR 0.69 (0.56–0.86)	PD-L1^+^ HR 0.56 (0.40–0.79); PD-L1^−^ HR 0.85 (0.62–1.18); interaction not significant (exploratory *p* = 0.08 and 0.35) [41]; *p* int = 0.08.
EV-302 [30,111]	First-line; EV + pembrolizumab vs. chemotherapy	22C3	OS HR 0.47 (0.38–0.58); ORR 67.7% vs. 44.4%	CPS ≥ 10 HR 0.49 (0.37–0.66); CPS < 10 HR 0.44 (0.31–0.61); benefit independent of PD-L1; *p* int = 0.64.
CheckMate 901 [112]	First-line; nivolumab + gemcitabine–cisplatin vs. chemotherapy	28-8	OS HR 0.78 (0.63–0.96); PFS HR 0.72 (0.59–0.88)	PD-L1 ≥ 1% (36%): OS HR 0.75 (0.53–1.06), PFS HR 0.60 (0.41–0.81); benefit irrespective of PD-L1 per the investigators; PD-L1 < 1% not reported.
CheckMate 901, nivolumab + ipilimumab [113]	First-line, cisplatin-ineligible; nivolumab + ipilimumab vs. gemcitabine–carboplatin (congress abstract)	28-8	OS HR 0.79 (98.27% CI 0.61–1.01; *p* = 0.0245 against a boundary of 0.0173); not significant	PD-L1 ≥ 1% (co-primary; *n* = 250, cisplatin-eligible and -ineligible): OS HR 0.87, not significant (*p* = 0.36); second failed PD-L1-selected co-primary comparison after DANUBE; PD-L1 < 1% not reported.
CheckMate 274 [31,36]	Adjuvant; nivolumab vs. placebo	28-8	DFS HR 0.70 (0.57–0.86) at primary analysis; 0.74 (0.61–0.90) at 5 years	Primary analysis: PD-L1 ≥ 1% (co-primary) HR 0.55 (0.39–0.77); PD-L1 < 1% (exploratory, unstratified; *n* = 414) HR 0.83 (0.64–1.08); *p* int = 0.06. At 5 years: PD-L1 ≥ 1% HR 0.58 (0.42–0.79); PD-L1 < 1% given only in the supplementary appendix. Basis for EU label restriction.
AMBASSADOR [37]	Adjuvant; pembrolizumab vs. observation	22C3	DFS HR 0.73 (0.59–0.90); interim OS HR 0.98 (0.76–1.26)	PD-L1^+^ (CPS ≥ 10) HR 0.81 (0.61–1.08); PD-L1^−^ HR 0.71 (0.53–0.95); declared prognostic, not predictive; *p* int = 0.53.
IMvigor010 [38]	Adjuvant; atezolizumab vs. observation	SP142	DFS HR 0.89 (0.74–1.08)	IC2/3 HR 1.01 (0.76–1.35); IC0/1 HR 0.85 (0.66–1.10); prognostic, not predictive; *p* int = 0.38.
IMvigor011 [8]	Adjuvant, ctDNA-positive after cystectomy; atezolizumab vs. placebo	SP142	DFS HR 0.64 (0.47–0.87); OS HR 0.59 (0.39–0.90)	DFS benefit reported as larger in IC2/3 than in IC0/1 (stratification factor); estimates in the supplementary appendix only; no interaction test.
NIAGARA [78]	Perioperative durvalumab + NAC vs. NAC	SP263	EFS HR 0.68 (0.56–0.82); OS HR 0.75 (0.59–0.93)	EFS benefit consistent in PD-L1-high (*n* = 777) and low/none (*n* = 286) subgroups; inter-laboratory inconsistency in the TC/IC 25% algorithm led the investigators to re-analyze by TC 1% and 25%, with consistent results.
KEYNOTE-905/EV-303 [79]	Perioperative, cisplatin-ineligible; EV + pembrolizumab with cystectomy vs. cystectomy alone	22C3	EFS HR 0.40 (0.28–0.57); OS HR 0.50 (0.33–0.74)	EFS and OS benefit reported as consistent in CPS ≥ 10 and CPS < 10 subgroups (forest plots); no interaction test.
KEYNOTE-B15/EV-304 [80]	Perioperative, cisplatin-eligible; EV + pembrolizumab vs. neoadjuvant gemcitabine–cisplatin	22C3	EFS HR 0.53 (0.41–0.70); OS HR 0.65 (0.48–0.89)	EFS benefit reported as consistent regardless of CPS (forest plot); no interaction test.
VOLGA [88]	Perioperative, cisplatin-ineligible; durvalumab + neoadjuvant EV (±tremelimumab) vs. cystectomy alone	SP263	Durvalumab + EV: EFS HR 0.51 (0.38–0.67); OS HR 0.58 (0.40–0.83)	Durvalumab + EV: PD-L1-high (52%) EFS HR 0.58 (0.39–0.86); PD-L1-low or negative HR 0.46 (0.31–0.67); *p* int = 0.41 (from the congress presentation).
KEYNOTE-866 [89]	Perioperative, cisplatin-eligible; pembrolizumab + gemcitabine–cisplatin vs. placebo + gemcitabine–cisplatin (congress abstract)	22C3	EFS HR 0.88 (0.71–1.10), not significant; OS HR 1.00 (0.79–1.27)	CPS ≥ 10 EFS HR 0.87 (0.65–1.19); CPS < 10 HR 0.94 (0.68–1.28); *p* int = 0.73; stratification factor; no benefiting subgroup.
CA017-078/ENERGIZE [90]	Perioperative, cisplatin-eligible; nivolumab + gemcitabine–cisplatin then adjuvant nivolumab vs. gemcitabine–cisplatin (congress abstract)	28-8	EFS HR 0.94 (95.74% CI 0.71–1.25), not significant; OS HR 0.88 (0.64–1.21)	PD-L1 ≥ 1% EFS HR 0.69 (0.45–1.04); PD-L1 < 1% HR 1.17 (0.81–1.70); *p* int = 0.06; pCR 46.4% vs. 35.5% and 33.3% vs. 24.3%.
CREST (Section 10)	BCG-naive high-risk NMIBC; sasanlimab + BCG vs. BCG	SP263	EFS HR 0.68 (0.49–0.94)	PD-L1-high in about one-fifth of analyzed tumors; baseline PD-L1 status not associated with the EFS benefit (exploratory; congress abstract).

BSC, best supportive care; CPS, combined positive score; DFS, disease-free survival; EFS, event-free survival; EU, European Union; EV, enfortumab vedotin; HR, hazard ratio; IC, immune cells; ITT, intention-to-treat; NAC, neoadjuvant chemotherapy; ORR, objective response rate; OS, overall survival; PD-L1, programmed death-ligand 1; PFS, progression-free survival; *p* int, approximate interaction *p* value calculated from the published subgroup hazard ratios and confidence intervals (Section 2.1), post hoc, underpowered and unadjusted for multiplicity. Confidence intervals are 95%. CheckMate 274 primary-analysis hazard ratios are given with the 95% confidence intervals reported in the United States prescribing information and the FDA approval announcement [47,49]; the primary publication reports 98.22% and 98.72% confidence intervals for the two co-primary comparisons [36]. Assays and thresholds differ between trials, so estimates are not directly comparable. The monotherapy comparisons in KEYNOTE-361 and IMvigor130 were exploratory because earlier hypotheses in the testing hierarchy were not met. For VOLGA, KEYNOTE-866 and CA017-078/ENERGIZE, reported as congress presentations at the time of writing, the assay is that of the sponsor’s program and the PD-L1 subgroup estimates are taken from the presentations and are provisional; for the nivolumab–ipilimumab comparison of CheckMate 901 the confidence interval of the PD-L1-positive estimate was not consistently reported in the congress coverage and is omitted. Phase 3 trials with a non-ICI comparator that had reported no PD-L1-stratified outcome (POTOMAC and ALBAN) or no results (NILE, KEYNOTE-676, KEYNOTE-992 and SWOG S1806) at the time of writing are not listed. pCR, pathological complete response.

**Table 4:** Level of evidence and implementation status of candidate predictors of immune checkpoint inhibitor benefit in bladder urothelial carcinoma, October 2026.

Biomarker	Strongest Supporting Evidence (Design Category, Section 2.2)	Implementation Status (Jurisdiction-Dependent)	Recommendation
Post-cystectomy ctDNA (tumor-informed)	Category A: prospective RCT with an enrichment design (IMvigor011), level I for treatment of ctDNA^+^ patients; meets the enrichment standard, not the predictive or strategy standard (Section 2.1). ctDNA^−^ patients: observed cohort of IMvigor011 and the exploratory strata of IMvigor010 (category B, criteria incompletely met) and CheckMate 274 (category D), with approximate interaction *p* = 0.004 and 0.03 for DFS and 0.016 and 0.15 for OS, unadjusted for multiplicity; no randomized evidence.	Actionable for selecting adjuvant atezolizumab in the United States (FDA companion diagnostic; NCCN Category 1); not authorized in the EU; no EAU recommendation.	Where available, perform serial testing after cystectomy in ICI-naive patients and treat with adjuvant atezolizumab on positivity within 12 months; observation of persistently negative patients is reasonable but rests on an observed cohort and on exploratory subgroup analyses of randomized trials, has not been compared with adjuvant nivolumab, and should be presented to patients as a judgment under uncertainty; not validated after perioperative ICI.
PD-L1 IHC	Category A for the enrichment question: positive co-primary PD-L1-positive populations in KEYNOTE-045, JAVELIN Bladder 100 and CheckMate 274, meeting the enrichment standard for three agent-and-assay pairs, but in trials positive in all patients as well; failed prespecified tests in IMvigor211 and DANUBE. PD-L1-negative strata exploratory (category D) in every trial; no prespecified interaction test, no significant approximate interaction (*p* = 0.06 to 0.73 in nine trials) and no completed randomized comparison showing that PD-L1-negative patients do not benefit; meets neither the predictive nor the strategy standard (prognostic association present).	Label-dependent/regional (EU).	Do not use to select combination, maintenance or later-line therapy. Test where a label requires it: in the EU, adjuvant nivolumab, and first-line pembrolizumab or atezolizumab monotherapy in cisplatin-ineligible patients.
Tumor mutational burden	Category D: exploratory analyses of single-arm and randomized trials (approximate interaction *p* = 0.06 in IMvigor211, 0.24 in KEYNOTE-361); tissue-agnostic accelerated approval from a trial containing no urothelial cohort.	Investigational	Do not use to select or withhold ICI in bladder cancer.
Consensus molecular subtypes	Category D: inconsistent associations in single-arm correlative studies.	Investigational	Research use only; no standardized clinical assay.
T-effector and TGF-β signatures; tertiary lymphoid structures	Category D: IMvigor210 and single-arm neoadjuvant correlative analyses; phenotype-dependent, inconsistent associations.	Investigational	Research use only; mechanistically informative but not a validated assay.
DNA damage response alterations	Category D: retrospective ICI-treated cohorts without comparator; confounded by platinum sensitivity.	Investigational	Do not use to select ICI therapy.
FGFR3 alterations	Category D: retrospective and real-world ICI-treated cohorts showing no consistent difference in outcome, unable to separate prognostic from predictive effects; THOR cohort 2 (erdafitinib not superior to pembrolizumab) enrolled FGFR-altered patients only.	Investigational	Test to identify erdafitinib candidates; must not be used to withhold ICI.
dMMR/MSI-H	Pooled single-arm trials (tissue-agnostic FDA approval, regular since 2023); abstract-level data in urothelial carcinoma; uncommon in urothelial carcinoma and mostly upper tract.	Label-dependent/regional (United States); rare	ICI is indicated under the tissue-agnostic FDA label; assess for Lynch syndrome.
Clinical and hematological indices	Prognostic models from treated cohorts (category C/D); no interaction tests reported in randomized trials; prognostic, not predictive.	Not applicable: no label or guideline directs an ICI decision on them	Use for prognostic counseling and stratification, not for treatment selection.

ctDNA, circulating tumor DNA; dMMR, mismatch repair deficiency; EAU, European Association of Urology; EU, European Union; FDA, U.S. Food and Drug Administration; ICI, immune checkpoint inhibitor; IHC, immunohistochemistry; MSI-H, high microsatellite instability; NCCN, National Comprehensive Cancer Network; PD-L1, programmed death-ligand 1; RCT, randomized controlled trial; TGF-β, transforming growth factor beta. Design categories follow Simon, Paik and Hayes [14] (Section 2.2). Implementation status describes whether the test can currently direct an ICI decision and depends on regulatory and guideline status, which differ between jurisdictions; it is not a grading of evidence. The tiers are those of Figure 4: actionable, a guideline recommends the test to direct an ICI decision and the resulting use is authorized; label-dependent/regional, a regular approval in at least one jurisdiction makes access to an ICI depend on the test; investigational, neither applies.

## 10. Non-Muscle-Invasive Disease

In BCG-unresponsive high-risk NMIBC, KEYNOTE-057 evaluated single-agent pembrolizumab. In cohort A (carcinoma in situ with or without papillary disease), the three-month complete response rate was 41% (95% CI 31–51) with a median duration of response of 16.2 months, supporting regulatory approval [119]. Durability was limited: 18 of the 39 complete responders (46%) remained in complete response for at least twelve months, corresponding to fewer than one in five of all treated patients.

No validated predictive biomarker exists in this setting. PD-L1 expression did not reliably identify patients achieving complete response: complete responses were observed irrespective of tumor PD-L1 status, although the subgroups were too small for definitive conclusions [119]. Molecular correlates of resistance remain investigational. Urinary tumor DNA is an attractive candidate given direct contact between urine and the tumor, and the NIAGARA urinary tumor DNA analysis provides encouraging proof of principle in muscle-invasive disease [73], but no assay is ready for clinical use in NMIBC.

ICIs have now also been tested in combination with BCG in BCG-naive high-risk disease, in three phase 3 trials that enrolled without biomarker selection. Durvalumab plus BCG induction and maintenance improved disease-free survival over BCG alone in POTOMAC (HR 0.68, 95% CI 0.50–0.93) [120], and subcutaneous sasanlimab plus BCG improved event-free survival in CREST (HR 0.68, 95% CI 0.49–0.94) [121], whereas atezolizumab plus BCG did not improve event-free survival in ALBAN (HR 0.98, 95% CI 0.71–1.36) [122]. Regulators have again read the same data differently: in May 2026 the FDA approved durvalumab with BCG for BCG-naive high-risk NMIBC [123], whereas the corresponding European application was withdrawn in June 2026 after the EMA’s provisional view that the benefits did not outweigh the risks [124]. The gains come at a cost in toxicity: treatment-related adverse events of grade 3 or 4 occurred in 21% versus 4% of patients in POTOMAC, and of grade 3 or higher in 29.1% versus 6.3% in CREST [120,121]. The case for a predictive biomarker is therefore, if anything, stronger here than in advanced disease. None is available. CREST measured PD-L1 with the SP263 assay and classified about one-fifth of analyzed tumors as PD-L1-high [121]; in an exploratory biomarker analysis presented as a congress abstract, baseline PD-L1 status was not associated with the event-free survival benefit of sasanlimab [125]. Results of KEYNOTE-676, which tests pembrolizumab with BCG, had not been reported at the time of writing.

## 11. Discussion

### 11.1. Why PD-L1 Did Not Become a Predictive Biomarker

PD-L1 has not become a predictive biomarker in urothelial carcinoma, and the reasons are overdetermined. We use “failure” in a specific sense: failure to meet the predictive standard of Section 2.1 and to be useful for selecting patients, not the absence of any association with outcome, and not the absence of efficacy in PD-L1-positive patients, which three co-primary comparisons established (the enrichment standard; Section 3.4 and Section 3.5). An association is present, and is partly prognostic, in the trials with more favorable estimates in PD-L1-high patients (KEYNOTE-045, JAVELIN Bladder 100 and CheckMate 274); what is absent is a replicated interaction, and what is present in the other trials is the opposite pattern. Analytically, four assays measuring different compartments with different algorithms cannot yield a portable classification. Biologically, PD-L1 expression is dynamic, spatially heterogeneous and modified by prior therapy, so that a single archival block gives an unreliable snapshot. Conceptually, PD-L1 marks one node in a redundant network of immune evasion, and its presence does not guarantee dependence on that axis, nor does its absence exclude response.

There is also a methodological lesson. PD-L1-defined populations entered the registrational trials on the strength of response-rate enrichment in single-arm studies [3,4,6], and they were built into the randomized designs as co-primary or hierarchical populations rather than tested through a treatment-by-biomarker interaction. Where the PD-L1-defined population was tested first, as in IMvigor211, or as a co-primary population for monotherapy, as in DANUBE, the comparison failed; where it was co-primary in a positive trial, as in KEYNOTE-045, JAVELIN Bladder 100 and Check-Mate 274, the trial was positive in all patients as well, so the contribution of the marker could not be isolated. Those three comparisons are category A analyses for the enrichment question and meet the enrichment standard of Section 2.1 (see Table 4 below), as IMvigor011 does for ctDNA; what they could not do, because the complementary strata were exploratory and no interaction test was prespecified, was show that the marker modified the effect or that PD-L1-negative patients should be treated differently. In no trial did PD-L1 turn a negative comparison in all patients into a positive one in the selected population, which is the circumstance in which an enrichment result changes a treatment decision. The first-line label restrictions of 2018 did rest on randomized data, but on interim signals of harm in PD-L1-low patients, not on a demonstration that PD-L1-high patients do better with an ICI than with chemotherapy. A decade of subgroup analyses has not resolved a question that a small number of marker-stratified trials, designed for the purpose, could have answered.

### 11.2. What ctDNA Has Shown, and What It Has Not

ctDNA has reached a point in clinical development that PD-L1 did not, and the contrast is instructive, although the two were not attempting the same thing and have not been tested in the same way. ctDNA measures residual disease burden rather than a putative mechanism of sensitivity, which makes it robust to the biological complexity that defeats expression-based markers; it also means that it selects patients who need adjuvant treatment, not patients whose tumors are sensitive to checkpoint blockade. It is quantitative and dynamic rather than a single static snapshot, and serial measurement adds to a single test: about 41% of the randomized patients in IMvigor011 were identified only on a later draw. Above all, its development followed a sound sequence—a prespecified exploratory hypothesis in IMvigor010, then a prospective randomized trial designed specifically around that hypothesis in IMvigor011, then regulatory and guideline adoption, so far in the United States.

It is worth stating plainly what IMvigor011 does and does not establish, by the three standards set out in Section 2.1. It meets the enrichment standard: among ctDNA-positive patients, adjuvant atezolizumab prolongs disease-free survival and, at an interim analysis tested within the hierarchy, overall survival compared with placebo, so ctDNA is a validated enrichment marker for that treatment (category A, level I evidence for that question). It does not meet the predictive standard: the persistently ctDNA-negative cohort was observed rather than randomized, so the trial demonstrates that these patients have a favorable untreated prognosis, not that they would derive no benefit from treatment, and it contains no test of a treatment-by-ctDNA interaction. Nor does it meet the strategy standard: ctDNA-guided treatment was compared with placebo in ctDNA-positive patients, not with adjuvant treatment of all high-risk patients, so the trial does not show that selecting by ctDNA is better than, or as good as, treating on pathological risk. Judged by the same standards, PD-L1 meets the enrichment standard for three agent-and-assay pairs and neither of the other two (Section 11.1), so the two biomarkers stand at the same level of the framework. The difference between them lies in what the marker-positive result meant for the treatment decision. Adjuvant atezolizumab had failed in the unselected population of IMvigor010, so the IMvigor011 result exists only because of the marker, the treatment cannot be offered without the test, and the test determines whether a patient is treated at all. The positive PD-L1 co-primary comparisons came from trials that were also positive in all randomized patients, so the marker was not needed to reach the decision; the one decision it has changed, the European restriction of adjuvant nivolumab, rests on the absence of established efficacy in an exploratory PD-L1-negative stratum rather than on evidence that the stratum does not benefit. The evidence that an ICI adds little or nothing in ctDNA-negative patients comes from the prespecified exploratory analysis of IMvigor010 (category B, criteria incompletely met) and the post hoc analysis of CheckMate 274 (category D), the same tier of evidence as the exploratory PD-L1-negative strata of the PD-L1 trials, not as their co-primary comparisons, and their confidence intervals (0.81–1.62 and 0.51–1.93) do not exclude a moderate benefit. Applying to these data the same approximate interaction test that we applied to PD-L1, for the endpoint that each trial reports by stratum, the ratio of hazard ratios between the ctDNA strata for disease-free survival was 0.51 (95% CI 0.32–0.81; *p* = 0.004) in IMvigor010 and 0.35 (95% CI 0.14–0.90; *p* = 0.03) in CheckMate 274, against *p* values of 0.06 to 0.73 for the nine PD-L1 comparisons (Table 3); for overall survival, which both ctDNA analyses also report by stratum, the values are *p* = 0.016 and *p* = 0.15. The IMvigor010 signal is numerically the strongest in this literature, but the comparison does not establish that the ctDNA evidence is stronger: none of the sixteen tests reported in this review was prespecified or adjusted for multiplicity, so that about 0.8 of them would be expected to reach *p* < 0.05 by chance and only the IMvigor010 disease-free survival test approaches a Bonferroni-corrected threshold; the CheckMate 274 test rests on 133 patients and is not significant for overall survival; and the three smallest PD-L1 values, 0.06 in CheckMate 274 and in CA017-078/ENERGIZE and 0.08 in JAVELIN Bladder 100, all in the direction of greater benefit in PD-L1-positive patients, are not far from the CheckMate 274 ctDNA value. Two further things set ctDNA apart. In the negative stratum of both trials the hazard ratio was close to or above one, not merely less favorable than in the positive stratum; and the prognosis of persistently negative patients is good enough that the absolute benefit of any adjuvant treatment could only be small. That combination makes observation with continued serial testing a reasonable option in this group, and it is the option that the FDA label and the NCCN guideline describe; but it is a judgment under uncertainty rather than a proven strategy, it has not been compared with adjuvant nivolumab given on pathological risk, and it should be presented to patients as such. The NIAGARA data (Section 5.4) sharpen the point: baseline ctDNA negativity did not identify patients without benefit from perioperative immunochemotherapy, so a negative test cannot be generalized into a rule for withholding an ICI outside the setting in which it was tested. Nor is the benefit in ctDNA-positive patients complete: most still relapse, and within that group the search for a predictor of sensitivity to ICI begins again.

### 11.3. Implementation: Cost, Turnaround Time, Access and the Consequences of Testing

The two biomarkers at the center of this review differ as much in logistics as in evidence. PD-L1 immunohistochemistry is inexpensive, is performed in any pathology laboratory on routine sections and returns a result within days; its weaknesses are analytical and biological, not logistical. Tumor-informed ctDNA testing is the reverse. The assay used in IMvigor011 requires whole-exome sequencing of the resected tumor and matched normal DNA to design a patient-specific panel before the first plasma sample can be analyzed, so that the first result cannot be available before surgery by design. In the IMvigor010 analysis the turnaround time was two to three weeks for the first plasma sample, including tissue sequencing and assay design, and about one week for each subsequent sample, which reuses the panel [7,8,62,63]. The IMvigor011 schedule, which the FDA label reproduces, requires a plasma sample every six weeks for a year, between seven and nine assays per patient depending on the time of enrollment, and treatment is triggered only by a positive result, so that about two-thirds of tested patients will not receive the drug [8,75]. Testing is centralized in a single commercial laboratory in the United States, which raises questions of sample logistics, data governance and continuity of supply for health systems elsewhere, and the test has no European authorization as a companion diagnostic for this indication because the indication itself does not exist [46]. A cost-effectiveness model based on IMvigor011, from the perspective of a US payer and over a five-year horizon, found the ctDNA-guided strategy dominant over its standard-of-care comparator, with 3.05 versus 2.92 quality-adjusted life years at a cost of $78,796 versus $131,601, and reported the result as robust in sensitivity analyses [77]; the published abstract defines the comparator only as standard of care and gives no threshold of ctDNA-positivity prevalence at which the conclusion would change, so that the saving is plausibly, but not demonstrably, attributable to drug not given to ctDNA-negative patients. Whether these conclusions transfer to European health systems with different drug prices, test prices and comparators (adjuvant nivolumab rather than observation) is unknown, and to our knowledge no European analysis has been published.

The consequences of a biomarker-guided decision also differ in kind from those of an unselected one. A false-negative ctDNA result delays treatment until radiographic recurrence, when adjuvant therapy is no longer an option; the IMvigor011 data put the two-year risk of relapse or death among persistently negative patients at about 12%, and about one-third of the patients who became positive did so too late to be randomized [8]. A false-positive result would expose a patient to a year of immunotherapy without residual disease, which is rare with a tumor-informed assay but not impossible. For PD-L1, the consequence of misclassification is confined to the settings in which a label depends on it. In the European Union, a patient whose tumor is PD-L1-negative by the 28-8 assay cannot receive adjuvant nivolumab, although the CheckMate 274 data do not exclude benefit in that group, and a different assay might have classified the same tumor as positive (Section 3.2). Serial testing also carries a burden that unselected treatment does not, in repeated venepuncture, waiting for results and the anxiety of a positive test, and these should enter the discussion with patients alongside the toxicity figures in Section 5.3. Finally, access differs between health systems and within them. Outside the United States, and in resource-limited settings generally, the IMvigor011 strategy is not currently deliverable, and adjuvant nivolumab given on pathological risk remains the practicable alternative, as the EAU guideline recognizes [53]. The ESMO Precision Medicine Working Group’s 2022 recommendation that ctDNA-based residual-disease testing should not direct treatment outside clinical trials [92] predates IMvigor011; the trial answers the recommendation’s evidentiary objection for one decision, but not its concerns about assay standardization and generalizability, which remain (Section 5.8).

### 11.4. Limitations

This review is narrative. Although the randomized evidence was sought as a census and discordant data were deliberately presented, study selection was not governed by a registered protocol and selection bias cannot be excluded, and no formal risk-of-bias instrument was applied (Section 2.2 and Section 2.3). The approximate interaction tests that we report are post hoc calculations from published estimates, not prespecified analyses; they are underpowered and assume independence of the strata, and they are offered to make explicit what the subgroup data can and cannot show; sixteen are reported, none is adjusted for multiplicity, the endpoint tested is the one each trial reports by stratum, and the results are descriptive and are not used to rank the biomarkers (Section 2.1). The three evidentiary standards of Section 2.1 are our operationalization of published design criteria, and other reasonable definitions exist; whatever their merits, we have applied them identically to both biomarkers. Several key analyses, particularly the NIAGARA ctDNA and urinary tumor DNA datasets, the CREST biomarker analysis, the EV-302 Nectin-4 analysis and the PD-L1 subgroups of VOLGA, KEYNOTE-866 and CA017-078/ENERGIZE, are available only as congress abstracts and may change on full publication. Effect estimates drawn from different trials are not directly comparable because populations, comparators, assays and thresholds differ, and the subgroup hazard ratios quoted here are mostly exploratory and unadjusted for multiplicity. The ctDNA evidence base, the only one in this review on which a randomized trial, a companion-diagnostic approval and a guideline recommendation all rest, is also the newest: follow-up of the randomized IMvigor011 cohort is short and its overall survival result is an interim one, the outcomes of its ctDNA-negative patients derive from an observed cohort, the trial relied almost entirely on a single commercial platform, and its comparator was placebo rather than adjuvant nivolumab. Finally, the field is moving quickly. The perioperative regimens described in Section 5.7 will progressively reduce the proportion of post-cystectomy patients who are ICI-naive, the group to which the IMvigor011 result and the NCCN recommendation apply, and the regulatory positions summarized here, which differ between the United States and Europe, are those of 24 October 2026.

### 11.5. Future Directions

The level of evidence and the implementation status of each candidate predictor, as of October 2026, are summarized in Table 4. Four priorities follow from this analysis. First, ctDNA-guided strategies should be tested prospectively in the post-perioperative-immunotherapy population, where current evidence does not apply and where the NIAGARA data suggest that ctDNA negativity does not exclude benefit. Second, treatment at ctDNA positivity should be compared directly with adjuvant therapy given on pathological risk alone, and observation of ctDNA-negative patients should be tested by randomization, as MODERN is designed to do [91]. Third, composite biomarkers combining ctDNA with tumor-intrinsic features merit evaluation: the IMvigor010 observation that ctDNA-positive and TMB-high patients showed a disease-free survival hazard ratio of 0.34, an exploratory finding in an unplanned subgroup of 69 patients, suggests that residual disease burden and tumor immunogenicity may carry complementary information [7]. Fourth, and most fundamentally, future biomarker development in this disease should be embedded prospectively in randomized trials with marker-stratified designs and prespecified interaction tests. The PD-L1 experience demonstrates the cost of the alternative.

## 12. Conclusions

After a decade of intensive investigation, one biomarker has become the first on whose result a randomized trial, a companion-diagnostic approval and a guideline recommendation all turn in directing the use of immune checkpoint inhibitors in urothelial carcinoma of the bladder, and it does so by detecting residual disease, not by predicting sensitivity to treatment. Serial tumor-informed circulating tumor DNA testing after radical cystectomy identifies patients who derive significant disease-free and overall survival benefit from adjuvant atezolizumab, and identifies a large complementary group with favorable untreated outcomes who may reasonably be observed, although that group was not randomized, about one in eight of them relapsed or died within two years, and a benefit of treatment in them has not been excluded. This position is supported by a prospective randomized enrichment trial, an FDA-approved companion diagnostic and a Category 1 NCCN recommendation; it is not yet reflected in European labeling or in the EAU guideline, and it applies to patients who have not received perioperative checkpoint inhibition, a group that is becoming smaller.

PD-L1 immunohistochemistry, despite its historical prominence, should not be used to choose between immunotherapy-containing regimens in metastatic bladder cancer. Its assays are not interchangeable; its randomized record is inconsistent and includes failure of the prespecified tests in the PD-L1-selected populations of IMvigor211 and DANUBE; its three positive co-primary comparisons (KEYNOTE-045, JAVELIN Bladder 100 and CheckMate 274) came from trials that were positive in all patients; no trial has shown a significant treatment-by-PD-L1 interaction, the exploratory PD-L1-negative strata do not exclude benefit; and the current first-line standard of care is effective irrespective of it. Its remaining influence is regulatory: in the European Union it still governs adjuvant nivolumab and first-line monotherapy in cisplatin-ineligible patients. Tumor mutational burden, molecular subtypes, tumor-microenvironment signatures, DNA damage response alterations and FGFR3 status remain investigational and should not be used to withhold immunotherapy from any patient.

The broader lesson extends beyond bladder cancer. A biomarker earns clinical authority not through mechanistic plausibility or regulatory momentum, but through prospective randomized evidence, and the three standards of Section 2.1 say what that evidence can and cannot support. By those standards, ctDNA and PD-L1 are in the same position: each meets the enrichment standard, ctDNA for one decision and PD-L1 for three agent-and-assay pairs, and neither meets the predictive standard, because the patients each would spare treatment have not been randomized, or the strategy standard, because marker-guided management has not been compared with unguided management. What separates them is that the ctDNA result was necessary for the decision of whether to start adjuvant atezolizumab in an ICI-naive patient after cystectomy, in a setting where the same treatment had failed in an unselected population, whereas no PD-L1 result has turned a negative comparison into a positive one; the closest PD-L1 has come to changing a decision on its own, namely the avoidance of first-line monotherapy in tumors with low expression, has been in interim analyses.

## Figures and Tables

**Table 1:** Registrational PD-L1 immunohistochemistry assays in urothelial carcinoma and their scoring algorithms.

Antibody Clone	Platform	Scoring Algorithm	Threshold	Associated Agent
SP142	Ventana BenchMark	Immune cells: % of tumor area occupied by PD-L1^+^ immune cells	IC2/3 (≥ 5%)	Atezolizumab
22C3	Dako Autostainer Link 48	Combined positive score: PD-L1^+^ tumor and immune cells ÷ viable tumor cells × 100	CPS ≥ 10	Pembrolizumab
SP263	Ventana BenchMark	Tumor cells ≥ 25% with membrane staining; or ≥25% of immune cells staining if immune cells occupy >1% of tumor area; or 100% of immune cells staining if immune cells occupy 1% of tumor area	TC/IC ≥ 25%	Durvalumab; avelumab
28-8	Dako Autostainer Link 48	Tumor cells: % with membranous PD-L1 staining	TC ≥ 1%	Nivolumab

CPS, combined positive score; IC, immune cells; PD-L1, programmed death-ligand 1; TC, tumor cells. Assays are not interchangeable; see Section 3.2 and Figure 1b [20,21,22].

**Table 2:** Circulating tumor DNA evidence in muscle-invasive and advanced bladder urothelial carcinoma.

Study	Design	Population	Principal Finding	Evidence Level
IMvigor010 [7,65]	Phase 3, adjuvant atezolizumab vs. observation	581 evaluable; 214 (37%) ctDNA^+^	ctDNA^+^: DFS HR 0.58 (0.43–0.79); OS HR 0.59 (0.42–0.83). ctDNA^−^: DFS HR 1.14 (0.81–1.62); OS HR 1.31 (0.77–2.23)	Prespecified exploratory analysis of an RCT: analysis plan finalized before unblinding, but no predetermined sample size and no replication (category B, criteria incompletely met; level II at most); both ctDNA strata randomized; approximate interaction *p* = 0.004 (DFS) and 0.016 (OS)
IMvigor011 [8,67]	Phase 3, ctDNA-guided adjuvant atezolizumab vs. placebo	761 enrolled; 379 ctDNA^+^, of whom 250 randomized; 357 persistently ctDNA^−^ analyzed	ctDNA^+^: DFS HR 0.64 (0.47–0.87), 178 events; OS HR 0.59 (0.39–0.90), 96 of 130 planned deaths at an interim analysis. ctDNA^−^ untreated: 39 of 357 (11%) recurred or died at a median follow-up of 21.8 months; 24-month DFS 88.4%	Prospective RCT with an enrichment design (category A, level I for ctDNA^+^ patients): only ctDNA^+^ patients randomized; ctDNA^−^ patients observed, so no treatment effect is estimable in them and no interaction test is possible; meets the enrichment standard, not the predictive or strategy standard (Section 2.1)
CheckMate 274 [31]	Phase 3, adjuvant nivolumab vs. placebo	133 of 709 assessable; 54 (40.6%) ctDNA-detectable	ctDNA-detectable: DFS HR 0.35 (0.18–0.66); OS HR 0.41 (0.20–0.83). ctDNA-undetectable: DFS HR 0.99 (0.51–1.93); OS HR 0.87 (0.41–1.84)	Post hoc exploratory analysis of an RCT (category D); both ctDNA strata randomized; approximate interaction *p* = 0.03 (DFS) and 0.15 (OS)
TOMBOLA [72]	Single-arm phase 2; atezolizumab on ctDNA positivity after NAC and cystectomy	192 enrolled; 104 of 178 (58%) ctDNA^+^ within 2 years	Complete response in 50 of 84 evaluable patients (60%); 1-year RFS 97% if ctDNA^−^ vs. 76% if ctDNA^+^	Prospective, non-randomized (single-arm; cannot test prediction)
NIAGARA [66]	Phase 3, perioperative durvalumab + NAC vs. NAC	462 biomarker-evaluable: 460 at baseline, 422 before and 345 after cystectomy	EFS HR 0.73 (0.51–1.06) if baseline ctDNA^+^ and 0.45 (0.25–0.84) if ctDNA^−^. DFS HR 0.49 (0.28–0.84) if post-cystectomy ctDNA^−^ (*n* = 255); not calculable if ctDNA^+^ (*n* = 17)	Exploratory (category D); congress abstract; post-cystectomy strata are defined after randomization and are not a randomized comparison
KEYNOTE-361 [74]	Phase 3, first-line pembrolizumab vs. chemotherapy	130 pembrolizumab, 130 chemotherapy	ctDNA associated with pembrolizumab but not chemotherapy outcome; no independent value beyond RECIST	Retrospective exploratory (category D)

ctDNA, circulating tumor DNA; DFS, disease-free survival; EFS, event-free survival; HR, hazard ratio; NAC, neoadjuvant chemotherapy; OS, overall survival; RCT, randomized controlled trial; RECIST, Response Evaluation Criteria in Solid Tumors. Confidence intervals are 95%. The IMvigor010 overall survival hazard ratio is taken from the updated survival analysis [65]; the primary ctDNA analysis reported HR 0.59 (95% CI 0.41–0.86) [7]. Design categories follow Simon, Paik and Hayes [14] (Section 2.2); approximate interaction tests are calculated from the published subgroup estimates (Section 2.1) and are post hoc and unadjusted for multiplicity.

## Data Availability

The original contributions presented in this study are included in the article. Further inquiries can be directed to the corresponding authors.

## Abbreviations

The following abbreviations are used in this manuscript:

ADC	Antibody–drug conjugate
ASCO	American Society of Clinical Oncology
BCG	Bacillus Calmette–Guérin
BSC	Best supportive care
CHMP	Committee for Medicinal Products for Human Use
CI	Confidence interval
CPS	Combined positive score
ctDNA	Circulating tumor DNA
CTLA-4	Cytotoxic T-lymphocyte-associated protein 4
DDR	DNA damage response
DFS	Disease-free survival
dMMR	Mismatch repair deficiency
EAU	European Association of Urology
EFS	Event-free survival
EMA	European Medicines Agency
EU	European Union
EV	Enfortumab vedotin
EV+P	Enfortumab vedotin plus pembrolizumab
FDA	U.S. Food and Drug Administration
HER2	Human epidermal growth factor receptor 2
HR	Hazard ratio
IC	Tumor-infiltrating immune cells
ICC	Intraclass correlation coefficient
ICI	Immune checkpoint inhibitor
IHC	Immunohistochemistry
ITT	Intention-to-treat
la/mUC	Locally advanced or metastatic urothelial carcinoma
MIBC	Muscle-invasive bladder cancer
MRD	Molecular residual disease
MSI-H	High microsatellite instability
NAC	Neoadjuvant chemotherapy
NCCN	National Comprehensive Cancer Network
NMIBC	Non-muscle-invasive bladder cancer
ORR	Objective response rate
OS	Overall survival
PD-1	Programmed cell death protein 1
PD-L1	Programmed death-ligand 1
PFS	Progression-free survival
RCT	Randomized controlled trial
RECIST	Response Evaluation Criteria in Solid Tumors
RFS	Recurrence-free survival
SANRA	Scale for the Assessment of Narrative Review Articles
TC	Tumor cells
TCGA	The Cancer Genome Atlas
TGF-β	Transforming growth factor beta
TMB	Tumor mutational burden
